# Aripiprazole, but Not Olanzapine, Alters the Response to Oxidative Stress in Fao Cells by Reducing the Activation of Mitogen-Activated Protein Kinases (MAPKs) and Promoting Cell Survival

**DOI:** 10.3390/ijms252011119

**Published:** 2024-10-16

**Authors:** Barbara Kramar, Tinkara Pirc Marolt, Ayse Mine Yilmaz Goler, Dušan Šuput, Irina Milisav, María Monsalve

**Affiliations:** 1 Institute of Pathophysiology, Faculty of Medicine, University of Ljubljana, Zaloska 4, 1000 Ljubljana, Slovenia; 2 Genetic and Metabolic Diseases Research and Investigation Center, Marmara University, 34854 Istanbul, Turkey; 3 Department of Biochemistry, School of Medicine, Marmara University, 34854 Istanbul, Turkey; 4 Laboratory of Oxidative Stress Research, Faculty of Health Sciences, University of Ljubljana, Zdravstvena pot 5, 1000 Ljubljana, Slovenia; 5 Instituto de Investigaciones Biomédicas Sols-Morreale (CSIC-UAM), Arturo Duperier, 4, 28029 Madrid, Spain

**Keywords:** atypical antipsychotics (AAPs), secondary drug effects, schizophrenia, liver, mitochondria, oxidative stress (OS), MAPK

## Abstract

Prolonged use of atypical antipsychotics (AAPs) is commonly associated with increased cardiovascular disease risk. While weight gain and related health issues are generally considered the primary contributors to this risk, direct interference with mitochondrial bioenergetics, particularly in the liver where these drugs are metabolized, is emerging as an additional contributing factor. Here, we compared the effects of two AAPs with disparate metabolic profiles on the response of Fao hepatoma cells to oxidative stress: olanzapine (OLA), which is obesogenic, and aripiprazole (ARI), which is not. Results showed that cells treated with ARI exhibited resistance to H_2_O_2_-induced oxidative stress, while OLA treatment had the opposite effect. Despite enhanced survival, ARI-treated cells exhibited higher apoptotic rates than OLA-treated cells when exposed to H_2_O_2_. Gene expression analysis of pro- and anti-apoptotic factors revealed that ARI-treated cells had a generally blunted response to H_2_O_2_, contrasting with a heightened response in OLA-treated cells. This was further supported by the reduced activation of MAPKs and STAT3 in ARI-treated cells in response to H_2_O_2_, whereas OLA pre-treatment enhanced their activation. The loss of stress response in ARI-treated cells was consistent with the observed increase in the mitochondrial production of O_2_^•-^, a known desensitizing factor. The physiological relevance of O_2_^•-^ in ARI-treated cells was demonstrated by the increase in mitophagy flux, likely related to mitochondrial damage. Notably, OLA treatment protected proteasome activity in Fao cells exposed to H_2_O_2_, possibly due to the better preservation of stress signaling and mitochondrial function. In conclusion, this study highlights the underlying changes in cell physiology and mitochondrial function by AAPs. ARI de-sensitizes Fao cells to stress signaling, while OLA has the opposite effect. These findings contribute to our understanding of the metabolic risks associated with prolonged AAP use and may inform future therapeutic strategies.

## 1. Introduction

Atypical antipsychotics (AAPs) are the first-line treatment for schizophrenia and are also commonly prescribed for other psychiatric disorders, including bipolar disorder and treatment-resistant depression, as well as for the management of community-dwelling patients with Alzheimer’s disease, other forms of dementia [1], behavior issues associated with autism and intellectual disability [2], and other behavioral disorders including attention-deficit/hyperactivity disorder [3]. Global estimates of antipsychotic use in adults range from 1 to 2%, with a reported rate of 1.7% in the USA in 2020 [4]. Worryingly, the use among children and adolescents has been rapidly increasing [5]. AAPs are weak dopamine D2 receptor blockers with diverse additional pharmacological targets, including serotonin (5-HT) receptors [6]. The efficacy and side effects of AAPs are influenced by the ratio of 5-HT2A/D2 and 5-HT2C/D2 receptor affinities as well as their dissociation from the D2 receptor [7]. The mixed receptor profile of AAPs includes partial agonism at 5-HT1 receptors, antagonism at H1 and α2 receptors, muscarinic antagonism, positive allosterism, blockade of glycine transporters, and also brain-derived neurotrophic factor production [8].

AAP prescriptions are often life-long, making the management of side effects a crucial concern for both physicians and patients [9]. While some side effects are related to the direct pharmacological targets of AAPs in the CNS, many are drug-specific and likely result from off-target effects or interactions with cellular physiology. Common side effects of AAPs include metabolic complications such as dyslipidemia [10] and impaired glucose homeostasis [11]. These side effects, which are frequently accompanied by significant weight gain [12], can substantially increase the risk of cardiovascular disease (CVD) [13]. Given that individuals prescribed AAPs often have a higher baseline prevalence of metabolic syndrome (MetS) and CVD risk than the general population, the potential for these metabolic complications becomes even more concerning [14].

The mechanisms involved in the increased CVD risk have been extensively investigated but have yet to be clarified. While some factors may be patient-specific, the majority of the increased risk is attributed to the treatment itself [15]. It has been suggested that the sedative effect of some AAPs and the associated increase in food intake play a fundamental role [16]. However, other factors may also contribute; for example, AAPs can cause hepatotoxicity and liver inflammation [17]. As the liver is a central metabolic organ, this has a substantial impact on systemic metabolic homeostasis. Liver toxicity associated with AAPs is primarily related to their metabolism by P450 enzymes, which generates reactive oxygen species (ROS) and, in certain cases, reactive intermediate metabolites [18]. Recent research suggests that mitochondria may also contribute to increased ROS levels following AAP administration [19]. Due to their high hydrophobicity, AAPs can accumulate in biological membranes, including the mitochondrial inner membrane (IMM), potentially altering cell signaling and bioenergetics. Indeed, several AAPs have been found to directly disrupt mitochondrial bioenergetics [20]. Notably, mitochondrial damage and oxidative stress have also been implicated in neurodegenerative, neurodevelopmental, and behavior disorders [21]. Recent studies imply that defective mitochondrial homeostasis, including impaired mitochondria regulation of activity and turnover, has a negative impact on the ability of cells to respond to stressors and prevent the accumulation of cellular damage [22] and may also dysregulate the activation of cell death-related pathways. An early response to mitotoxins is the induction of mitochondrial fission, which enables the elimination of damaged mitochondria through mitophagy [23]. Fission has been shown to reduce both the oxidative capacity of mitochondria and their sensitivity to mitochondrial-dependent apoptosis, a process that is particularly relevant in cancer [24]. Interference with mitochondrial function can significantly disrupt cell physiology, nutrient response, oxidative stress management [25], and metabolic and cardiovascular health [26]. It can also negatively impact neurological homeostasis [23]. Given this background, it is crucial to evaluate the specific cellular effects and underlying mechanisms of AAPs to minimize their negative adverse effects.

Clozapine and olanzapine (OLA) are among the most concerning AAPs in terms of MetS risk [27]. OLA is the fourth most commonly prescribed AAP, accounting for ~10% of all AAP prescriptions. Importantly, type 2 diabetes (T2D) is not always strictly linked to adiposity (weight gain), and 25% of patients prescribed AAPs experience hyperglycemia without significant weight increase, often early in treatment. However, weight gain undoubtedly exacerbates this risk [28]. Despite its side effects, OLA is highly effective in reducing both positive and negative psychotic symptoms, significantly more than most other AAPs, and is associated with reductions in depressive episodes and improvements in social functioning [29]. These benefits contribute to its widespread use as one of the most commonly prescribed AAPs [30].

The precise mechanism of OLA action is unknown, as it exhibits diverse receptor affinities that may contribute to clinical and adverse effects. As mentioned earlier, its efficacy in schizophrenia may be due to a combination of mesolimbic dopamine D2 and serotonin 5-HT2A receptor antagonism [31]. OLA exhibits a higher affinity for serotonin 5-HT2A receptors than dopamine D2 receptors, with selectivity for dopamine receptor subtypes in the mesolimbic and mesocortical systems over the nigrostriatal and tuberoinfundibular systems. This may explain the reduced risk of extrapyramidal side effects (uncontrollable involuntary movements) and hyperprolactinemia. Antagonism of muscarinic, histaminic, and alpha-adrenergic receptors can lead to common side effects including dry mouth, micturition difficulty, constipation, weight gain, somnolence, dizziness, and hypotension [32]. OLA is well absorbed orally with 80% bioavailability despite undergoing first-pass metabolism, and reaches peak plasma concentrations after about 6 h. It is predominantly bound to albumin (90%) and α1-acid glycoprotein (77%), and follows linear kinetics with an elimination half-life of 21–54 h, achieving steady-state levels within a week [33]. In the liver, OLA is first metabolized to its 10- and 4′-N-glucuronides, 4′-N-desmethyl olanzapine [cytochrome P450(CYP)1A2] and olanzapine N-oxide [flavin monooxygenase 3], and then cleared. Metabolism to 2-hydroxymethyl olanzapine via CYP2D6 is a minor pathway. While OLA does not inhibit CYP isozymes [7], it has been shown to increase plasma transaminases levels, suggesting potential liver toxicity. This may be linked to its impact on whole-body metabolism, leading to liver steatosis and even non-alcoholic fatty liver disease in some cases [34].

Some in vitro studies have evaluated the impact of OLA on mitochondria and its role in redox homeostasis. For example, OLA has been shown to induce mitochondrial fission [35], downregulate electron transport chain (ETC) genes, and decrease mitochondrial enzyme activity, ATP synthesis, and the oxygen consumption rate (OCR) in circulating leukocytes of patients at increased risk for MetS [36]. Moreover, one study detected increased oxidative stress and glutathione depletion in hepatocytes treated with OLA, pointing to both mitochondrial toxicity and CYP450 activity [37]. Other studies on the mitochondrial toxicity of AAPs indicate that OLA has minimal or no direct mitochondrial toxicity, while other AAPs, in particular aripiprazole (ARI), have been shown to decrease the mitochondrial OCR [38] in liver cells. This effect may be related to the inhibition of ETC Complex I or Complex V activity [38,39]. While a complete understanding of the interaction between AAPs and mitochondria remains elusive, available data suggest that it may trigger mitochondrial dysfunction and lead to the development of MetS.

ARI is the second most commonly prescribed AAP, accounting for about 20% of total AAP prescriptions. It is a partial agonist of D2 receptors and 5-HT1A receptors, as well as antagonist 5-HT2A receptors [40]. Its unique pharmacological profile compared with other AAPs, including the absence of sedative and hyperphagic effects, makes it a preferred choice for patients at risk of or with MetS. ARI has been shown to induce debilitating extrapyramidal syndromes in 5–15% of patients, likely due to off-target effects. It is metabolized in the liver by cytochrome P450 (CYP)3A4 and 2D6 enzymes, and polymorphisms in these genes can reduce drug tolerance [41,42]. As reported by LiverTox^®^, updated in 2023, the hepatotoxicity likelihood score of ARI is categorized as “D” (possible rare cause of clinically apparent liver injury) [43]. Nevertheless, several cases related to ARI-induced liver injury have been reported [44], but large-scale epidemiological studies are lacking [45]. Notably, chronic feeding of adult *Drosophila melanogaster* with ARI has been shown to result in mitochondrial damage in the brain and thoracic muscles, leading to locomotor dysfunction [46], supporting the notion that ARI mitotoxicity occurs in vivo and can have significant pathological effects. In previous work, we also reported the molecular effects of ARI on mitochondria and oxidative stress in parenchymal liver cells [38,47].

It should be noted that schizophrenia patients have a higher incidence of metabolic syndrome even before the start of the treatment, and worse mitochondrial function than the general population [48]. Therefore, these subjects frequently show high ROS levels. Dysregulation of the gene expression of antioxidant proteins is a very common observation and presumably related with a relevant genetic component linked to schizophrenia [49]. In view of this, some authors have suggested a causal link between mitochondrial dysfunction and the risk of schizophrenia development, thus highlighting the potential risk of using mitotoxic medications [50].

Here, we investigate ARI and OLA secondary effects in liver cells aiming to elucidate the molecular basis of their metabolic side effects in patients. In order to test the changes in cell physiology induced by ARI and OLA after prolonged treatment, we could not use primary hepatocytes due to their short in vitro lifespan. Therefore, we used a rat hepatocarcinoma cell line, Fao, which retains metabolic plasticity and ROS sensing better than other hepatocyte cell lines [51,52,53]. Cells were dosed with the relevant physiological range of the drugs that increases the risk of toxicity (laboratory alert) [54] in addition to a higher concentration (6 μM) to account for the reported increased liver concentration compared with plasma [38]. We examined how single and daily treatments (repeated over several weeks) with ARI and OLA modified the cellular response to oxidative challenge. Our results imply that the mitotoxicity of ARI reduces cellular sensitivity to MAPK activation, leading to lower cell death rates after oxidative challenge.

## 2. Results

### 2.1. Mitochondrial Superoxide (O_2_^•-^)

**Figure 1:**
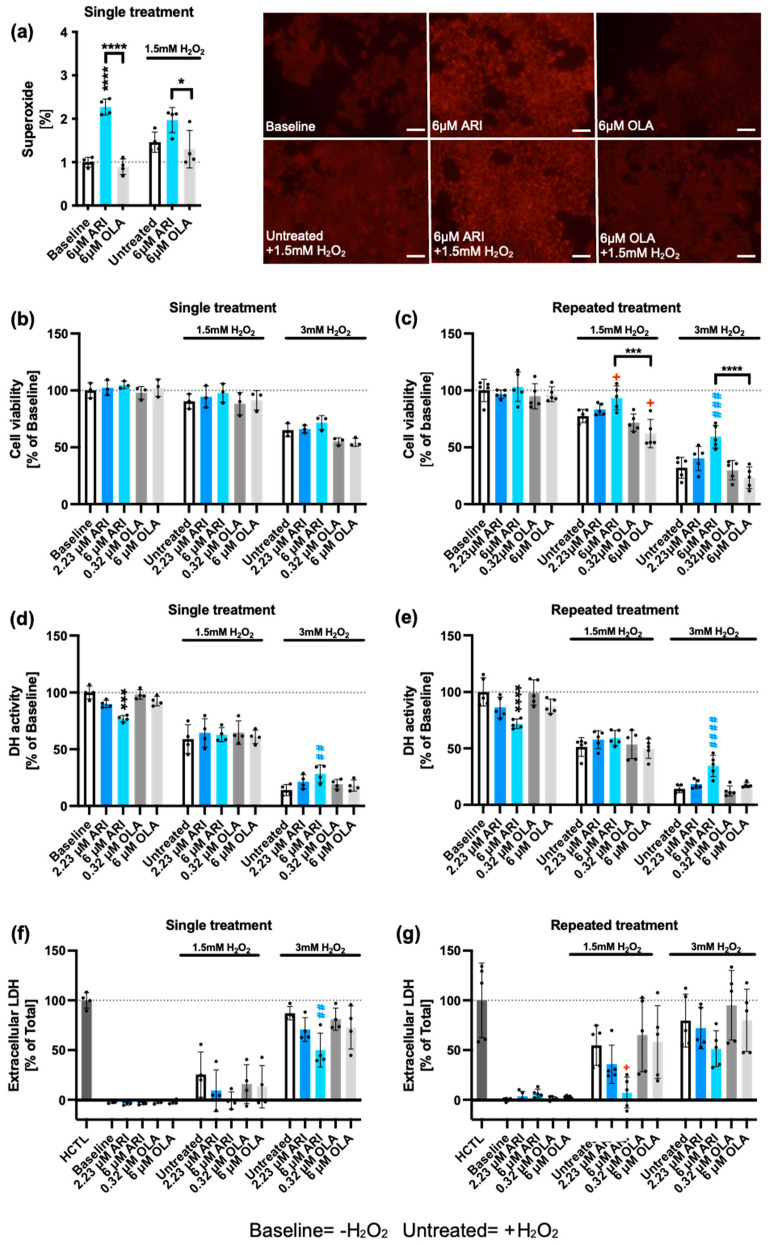
Superoxide production and cell viability. (**a**) Fao cells were stained with MitoSOX to evaluate mitochondrial O_2_^•-^ levels. Bar: 50 mm. Cell viability was assessed by neutral red (NR) accumulation (**b**,**c**), dehydrogenase (DH) activity by MTT reduction (**d**,**e**), and extracellular lactate dehydrogenase (LDH) by LDH release (**f**,**g**). Panels (**b**,**d**,**f**) represent data from single (24 h) ARI/OLA treatments, and panels (**c**,**e**,**g**) from repeated treatments. Baseline data of NR, MTT, and LDH assays (no H_2_O_2_ added) for both single and repeated treatments (**b**–**g**) are re-used under CC-BY4.0 license [56], as well as the NR assay results with 1.5 and 3 mM H_2_O_2_ in repeatedly treated cells (**c**) [38]. Each graph contains only simultaneously recorded data during the same experimental setup. The graphs show mean ± standard deviation (SD) values analyzed by one-way ANOVA followed by Dunnett’s test. Baseline ARI/OLA samples were compared with baseline control without H_2_O_2_ (*), 1.5 mM H_2_O_2_ ARI/OLA-treated cells were compared with 1.5 mM H_2_O_2_ untreated controls (+), and 3 mM H_2_O_2_ ARI/OLA-treated samples were compared with 3 mM H_2_O_2_ untreated controls (#). Tukey’s test was used to compare 6 µM ARI with 6 µM OLA samples (* above the line indicating the comparison). *^/+^ *p* ≤ 0.05, **^##^** *p* ≤ 0.01, ***^/###^ *p* ≤ 0.001, ****^/####^ *p* ≤ 0.0001. (**a**): *n* = 4; (**b**,**e**): *n* = 3; (**c**,**d**): *n* = 4; (**f**,**g**): *n* = 5. HCTL: “high control”, maximal release of LDH.

To examine the impact of ARI and OLA treatment on mitochondrial function in liver cells, we initially assessed mitochondrial O_2_^•-^ production using MitoSOX staining in ARI- and OLA-treated Fao cells. We found that O_2_^•-^ levels significantly increased after a single treatment with ARI, but not OLA, at a concentration of 6 μM, (Figure 1a), suggesting that ARI exerts considerable mitochondrial toxicity. This is consistent with previous observations that ARI at this dose and timing increases cellular H_2_O_2_ levels and decreases mitochondrial OCR [38].

ROS generally induce compensatory cellular responses, including the expression of antioxidants, dependent on the activation of redox-sensing transcription factors such as Nrf2 [55]. However, previous observations indicated that a single ARI treatment had no effect on the antioxidant capacity of Fao cells, as measured by superoxide dismutase (SOD), glutathione peroxidase (GPx), and catalase activities. However, continuous exposure to 6 μM ARI for 4–8 weeks led to a significant increase in GPx and catalase activities [38], suggesting that long-term ARI treatment modifies the cellular response to oxidative stress.

We evaluated this possibility by testing the response of Fao cells to a 3 h oxidative challenge with 1.5–3 mM H_2_O_2_ following repeated exposure to ARI or OLA for 4–8 weeks. We found that cells pre-treated with 6 μM ARI showed a significant increase in the activity of two antioxidants, SOD and catalase, the protein levels of the antioxidant Heme oxygenase-1, and the gene expression of another antioxidant, Sulfiredoxin-1 (*Srxn1*), and *Foxo3a*, a transcription factor that controls antioxidant gene expression. Similar treatment with OLA failed to induce any of these changes [47], indicating that ARI but not OLA enhances the resistance to oxidative stress upon oxidative challenge.

To determine whether the ARI-elicited changes in the response to oxidative challenge required long-term adaptation (repeated treatments), we tested whether single treatment with ARI/OLA also modified the response of Fao cells to oxidative challenge. Thus, we evaluated mitochondrial O_2_^•-^ levels using MitoSOX staining in Fao cells treated with 6 μM ARI or OLA followed by a 3 h challenge with 1.5 mM H_2_O_2_. We found that O_2_^•-^ levels were significantly higher in ARI-treated cells than in OLA-treated cells; and O_2_^•-^ levels in OLA-treated cells did not differ from controls, a result similar to that obtained in the presence of 1.5 mM H_2_O_2_ (Figure 1a).

### 2.2. Cellular Viability

Next, we aimed to determine whether these differences impacted the cellular viability of ARI/OLA-treated cells challenged with 1.5–3 mM H_2_O_2_ for 3 h. We first evaluated the lysosomal uptake of neutral red (NR), a metric to identify living cells. In single-treated cells, 3 mM H_2_O_2_, but not 1.5 mM H_2_O_2_, significantly decreased NR uptake, but no significant differences were observed between the treatment groups and controls exposed to H_2_O_2_ (Figure 1b). However, we previously observed [38] that in repeated treatments, following challenge with 1.5 and 3 mM H_2_O_2_, which decreased the cell viability of control cells, 6 μM ARI-treated cells had significantly higher NR levels than untreated controls (Figure 1c). Also, following the 1.5/3 mM H_2_O_2_ challenge, 6 μM OLA-treated cells showed significantly lesser survival than 6 μM ARI-treated cells (Figure 1c). These results imply that ARI-treated cells, although having higher ROS levels, are more resistant to oxidative stress-induced cell death. We further tested this notion using an alternative viability test, MTT, which measures cellular NAD(P)H dehydrogenase (DH) activities and is also indicative of the cellular metabolic status. In untreated controls, both 1.5 and 3 mM H_2_O_2_ significantly reduced DH activity. In cells exposed to 1.5 mM H_2_O_2_ following ARI treatment, no significant differences in MTT reduction were found between the groups (Figure 1d,e). However, in cells challenged with 3 mM H_2_O_2_, we observed, both for single- and repeatedly treated cells, a significantly higher survival rate for cells treated with 6 μM ARI over the untreated cells (Figure 1d,e), suggesting that ARI also enhances survival following single treatment (Figure 1d). Finally, we further tested cell viability using a lactate dehydrogenase (LDH) release assay, which measures cell rupture by determining LDH present in the extracellular medium. As expected, we found that a 1.5 and 3 mM H_2_O_2_ challenge increased LDH levels in untreated cells. Contrastingly, in single-treated cells, following a 3 mM H_2_O_2_ challenge, 6 μM ARI-treated cells had significantly lower LDH release than untreated controls (Figure 1f). In repeatedly treated cells, where both 1.5 and 3 mM H_2_O_2_ increased LDH release in untreated controls, 6 μM ARI-treated cells also had significantly lower LDH release than untreated controls following a 1.5/3 mM H_2_O_2_ challenge (Figure 1g). Overall, these data support the notion that ARI enhances cell survival following H_2_O_2_ challenge, whereas OLA has the opposite effect. Notably, at baseline (without H_2_O_2_), whereas 6 μM ARI treatment (in both single and repeated treatments) significantly reduced MTT activity, there was no corresponding decrease in NR or increase in LDH release, suggesting that this reduction in cell viability is possibly related to ARI mitotoxicity.

### 2.3. Apoptosis

**Figure 2:**
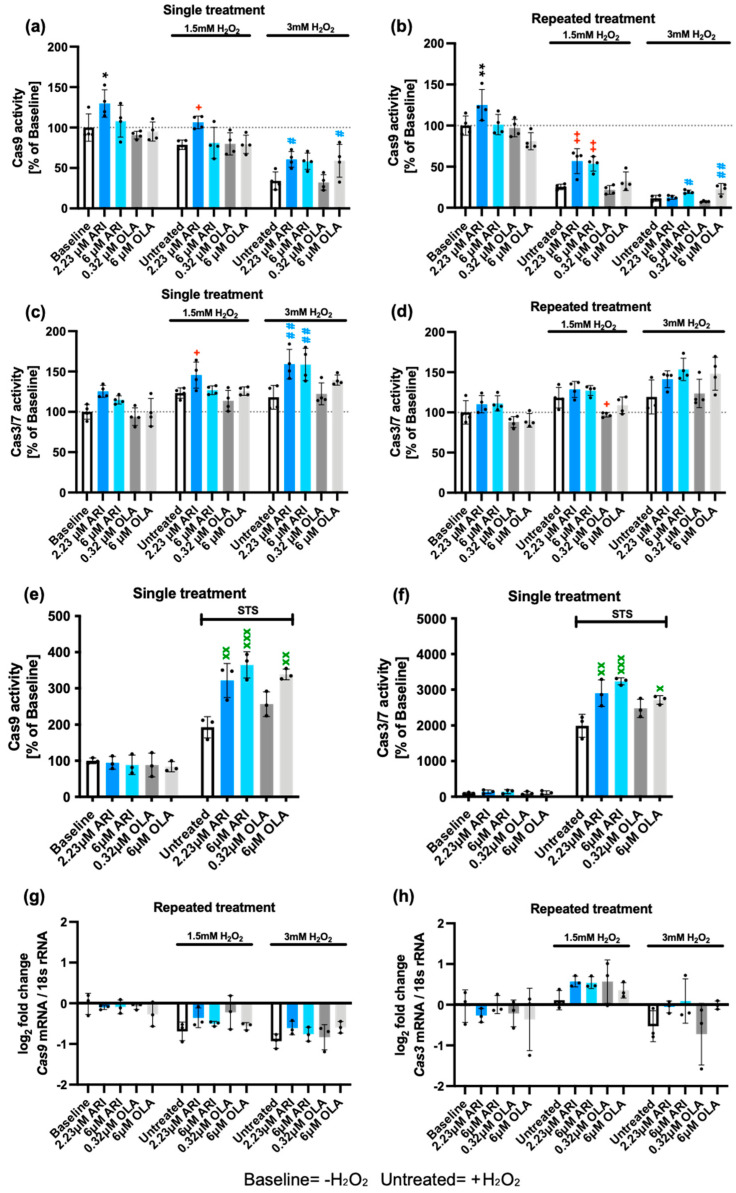
Caspase levels and activities. Caspase 9 activity in single- (**a**) and repeatedly (**b**) treated Fao cells and challenged with H_2_O_2_. Caspase 3/7 activity in single- (**c**) and repeatedly (**d**) treated Fao cells and challenged with H_2_O_2_. Caspase 9 and 3/7 activity baseline data (without H_2_O_2_) in single- and repeatedly treated cells (**a**–**d**) are re-used under CC-BY4.0 license [56]. Each graph contains only simultaneously recorded data during the same experimental setups. Caspase 9 (**e**) and 3/7 activity (**f**) in single-treated Fao cells challenged with staurosporine (STS). Gene expression analysis of *Caspase 9* (**g**) and *Caspase 3* genes (**h**). Data are presented as mean ± SD and analyzed by one-way ANOVA followed by Dunnett’s test, comparing samples within each group: untreated with treated, without H_2_O_2_ (*), untreated with treated, challenged with 1.5 mM H_2_O_2_ (+); untreated with treated, challenged with 3 mM H_2_O_2_ (#). Additionally, samples were analyzed with Tukey’s test to compare 6 µM ARI with 6 µM OLA (**e**,**f**): Dunnett’s test was used to test the comparison between the AAP treatments with STS to untreated controls with STS (x). *^/+/#/x^ *p* ≤ 0.05, **^/++/##/xx^ *p* ≤ 0.01, **^xxx^** *p* ≤ 0.001. (**a**–**d**): *n* = 4; (**e**–**h**): *n* = 3. Cas: Caspase.

Oxidative stress can lead to different types of cell death, including apoptosis, necroptosis, pyroptosis, and ferroptosis [57]. We next determined whether the activation of apoptosis in Fao cells, following H_2_O_2_ challenge, was affected by pre-treatment with ARI/OLA. We evaluated the activity of Caspase 9, which is mainly involved in the mitochondrial cell death pathway, and the final effectors of apoptosis, Caspase 3/7. Following both single- and repeated treatment with 2.23 μM ARI, Fao cells had significantly higher Caspase 9 activity than untreated cells (no H_2_O_2_ challenge) (Figure 2a,b). A similar increase has been previously observed [56]; however, it did not reach statistical significance possibly due to a fewer number of samples and/or the small variability in biological replicates. Even though Caspase 9 activity was overall lower in H_2_O_2_-treated cells than in baseline samples, we consistently found that both single and repeated treatment with 2.23 μM ARI also significantly maintained higher Caspase 9 activity compared to equally treated controls in 1.5 mM H_2_O_2_ challenged cells (Figure 2a,b). Nevertheless, when cells were challenged with 3 mM H_2_O_2_, both 6 μM ARI and 6 μM OLA in repeated treatments led to higher Caspase 9 levels than those of untreated cells (Figure 2a,b). These results indicate that the pro-survival effect of ARI is not related to a greater reduction in mitochondria-induced apoptosis. Indeed, the observation that ARI-treated cells consistently have higher Caspase 9 activity may relate to the higher levels of mitochondrial ROS and lower mitochondrial activity observed following this treatment.

We next evaluated Caspase 3/7 activity. Consistent with the results found for Caspase 9, we found that Fao cells exposed to a single treatment with 2.23 μM ARI maintained higher Caspase 3/7 levels than untreated controls following a challenge with 1.5 or 3 mM H_2_O_2_ (Figure 2c). Also, single treatment with 6 μM ARI resulted in significantly higher Caspase 3/7 activity than in untreated cells in response to a 3 mM H_2_O_2_ challenge (Figure 2c). Similar results were found in repeatedly treated cells, although the differences did not reach statistical significance (Figure 2d). By contrast, cells repeatedly treated with 0.32 μM OLA had significantly lower Caspase 3/7 activity than untreated controls following a challenge with 1.5 mM H_2_O_2_, which may indicate that OLA prevents apoptosis. Taken together, the results imply that ARI-treated cells are more sensitive to apoptotic cell death through the mitochondrial pathway, whereas OLA at low doses seems to be protective, although it can also promote apoptotic cell death in H_2_O_2_-treated cells at high doses. These observations may relate to the higher levels of mitochondrial ROS in ARI-treated cells and further suggest the concept that the pro-survival effects ARI are likely not related to the regulation of apoptosis.

To investigate whether the apoptotic response was specific to H_2_O_2_ exposure or a general response to other pro-apoptotic stimuli, cells were exposed to staurosporine (STS) following a single treatment with ARI/OLA. All treatments significantly increased Caspase activity over baseline levels. Additionally, both 2.23 and 6 μM ARI as well as 6 μM OLA increased Caspase 9 and Caspase 3/7 activity in response to STS treatment (Figure 2e,f). This confirms the general pro-apoptotic effect of ARI and OLA. While non-significant, both Caspase 9 and Caspase 3 levels were mostly higher for 6 μM ARI-treated cells than for equivalent OLA-treated cells following STS treatment, which aligns with previous data indicating that ARI is a stronger inducer of cell apoptosis than OLA.

We next determined whether Fao cells were responding to the treatments and the H_2_O_2_ challenge by modifying the gene expression of Caspases. In cells treated repeatedly with ARI/OLA, we found no significant changes in gene expression at baseline or after exposure to 1.5/3 mM H_2_O_2_. However, exposure to 1.5/3 mM H_2_O_2_ alone resulted in a small, significant downregulation of *Caspase 9* expression (Figure 2g), and 1.5 mM H_2_O_2_ induced a small, non-significant upregulation of *Caspase 3* (Figure 2h) in long-term AAP treated cells and their corresponding controls. Therefore, we concluded that ARI/OLA treatments do not affect *Caspase 9* and *3* gene expression.

In light of these findings, we investigated whether the observed disparities between ARI and OLA in their ability to induce apoptotic cell death might be attributable to alterations in gene expression/protein levels in other pro- or anti-apoptotic genes/proteins in repeatedly treated cells.

**Figure 3:**
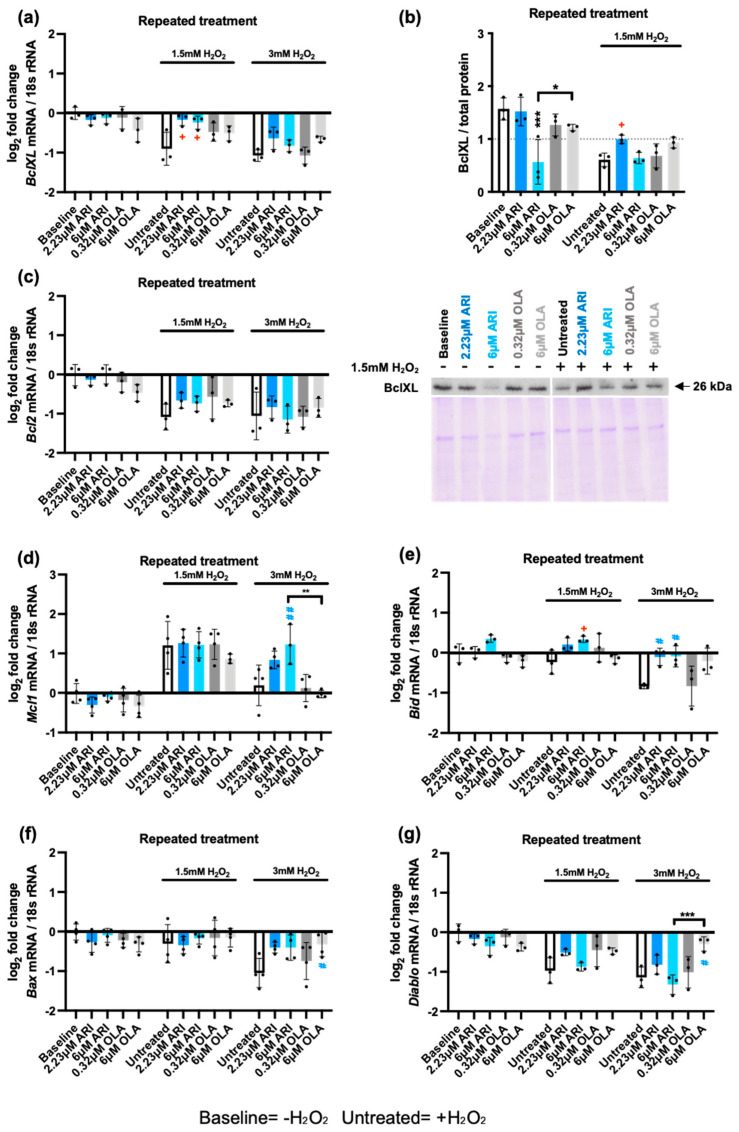
Pro- and anti-apoptotic factors. Log_2_ fold-change in mRNA expression levels of pro- and anti-apoptotic factors in repeatedly treated Fao cells: (**a**) *BclXL*, (**c**) *Bcl2*, (**d**), *Mcl1* (**e**) *Bid,* (**f**) *Bax* and (**g**) *Diablo*. (**b**) BclXL Western blot analysis and corresponding Coomassie blue stained gel (loading control). Data are presented as mean ± SD and analyzed by one-way ANOVA followed by Dunnett’s test, comparing samples within each group: untreated with treated, without H_2_O_2_ (*), untreated with treated, challenged with 1.5 mM H_2_O_2_ (+); untreated with treated, challenged with 3 mM H_2_O_2_ (#). Additionally, samples were analyzed with Tukey’s test to compare 6 µM ARI to 6 µM OLA samples (* above the line indicating the comparison). *^/+/#^ *p* ≤ 0.05, **^/##^ *p* ≤ 0.01, *** *p* ≤ 0.001. (**a**–**c**,**f**,**g**): *n* = 3; (**d**,**e**): *n* = 4.

We first tested the gene expression (Figure 3a) and protein levels (Figure 3b) of the anti-apoptotic BCL-2 family member BclXL [58] in repeatedly treated cells. BclXL protein and mRNA levels were reduced by H_2_O_2_ exposure in untreated cells. In response to AAP treatments, we found that ARI (6 μM) significantly decreased BclXL protein levels, but not gene expression, suggesting that this event was post-transcriptional. After exposure to 1.5 mM H_2_O_2_, we found that mRNA and protein levels of BclXL were higher in cells treated with 2.23 μM ARI than in untreated controls, indicating that these changes were transcriptionally regulated, with untreated cells being susceptible to BclXL downregulation and ARI-treated cells being more resistant. No significant differences were observed in response to OLA. These results suggest that if BclXL is an important factor for the greater capacity of ARI-treated cells to activate Caspase 9, it is likely to be related to the lower levels of BclXL in ARI-treated cells at baseline, prior to H_2_O_2_ exposure. They also suggest that it is the lack of responsiveness in ARI-treated cells that affects *Bclxl* transcriptional regulation.

Other anti-apoptotic BCL-2 family members were evaluated at the gene expression level. We found no differences in the baseline levels of *Bcl2* between AAP treatments, nor after challenge with 1.5/3 mM H_2_O_2_. However, H_2_O_2_ induced the downregulation of *Bcl2* at the two doses tested (Figure 3c), similar to what was observed for BclXL. In addition, we found no differences in the baseline levels of *Mcl1* between AAP treatments. After challenge with 1.5 mM H_2_O_2_, we found a significant upregulation of *Mcl1* in untreated cells and in APP treated cells. By contrast, 3 mM H_2_O_2_ exposure did not upregulate Mcl1 expression in untreated cells over baseline levels (Figure 3d). Of note, following challenge with 3 mM H_2_O_2_, cells pre-treated with 6 μM ARI had higher *Mcl1* expression than both untreated controls and 6 μM OLA-treated cells, suggesting that ARI maintains the cellular responsiveness to Mcl1 induction (Figure 3d). Overall, these results indicate that ARI-treated cells maintain higher anti-apoptotic gene expression levels than untreated or OLA-treated cells following H_2_O_2_ exposure, despite their higher caspase activity.

As apoptosis is governed to a large extent by the balance between pro- and anti-apoptotic factors, to better understand the significance of these findings, we analyzed the gene expression of two pro-apoptotic members of the Bcl-2 family, *Bid* (Figure 3e) and *Bax* (Figure 3f), and the pro-apoptotic factor *Diablo* [59] (Figure 3g). We found that *Bid* expression levels in cells were similar between the AAP treatments at baseline, but following exposure to 1.5 mM H_2_O_2_, we observed that cells treated with 6 μM ARI had higher *Bid* levels. Untreated cells exposed to 3 mM H_2_O_2_ had lower *Bid* levels than cells not exposed to H_2_O_2_, and cells treated with either 2.23 or 6 μM ARI had higher *Bid* levels than untreated and OLA-treated cells. *Bid* levels did not significantly differ between OLA-treated cells and untreated cells. Analysis of *Bax* and *Diablo* gene expression revealed that *Bax* levels were the lowest in 3 mM H_2_O_2_-treated cells, and both 1.5 and 3 mM H_2_O_2_ decreased *Diablo* expression in untreated cells. The tendency for decreased *Bax* and *Diablo* expression levels after H_2_O_2_ challenge was not significantly changed by ARI treatment, whereas cells pre-treated with 6 μM OLA maintained significantly higher expression levels of both *Bax* and *Diablo* when challenged with 3 mM H_2_O_2_. Furthermore, the higher OLA-dependent expression of *Diablo* was significant compared with ARI-dependent expression (Figure 3g). Overall, these results imply that ARI/OLA-treated cells have higher expression levels of both pro- and anti-apoptotic genes following H_2_O_2_ exposure, possibly making them more sensitive to both pro- and anti-apoptotic stimuli. The impact of ARI and OLA is different and gene-specific. Whether the overall pro-/anti- apoptotic balance is globally altered by the AAPs remains to be established.

**Figure 4:**
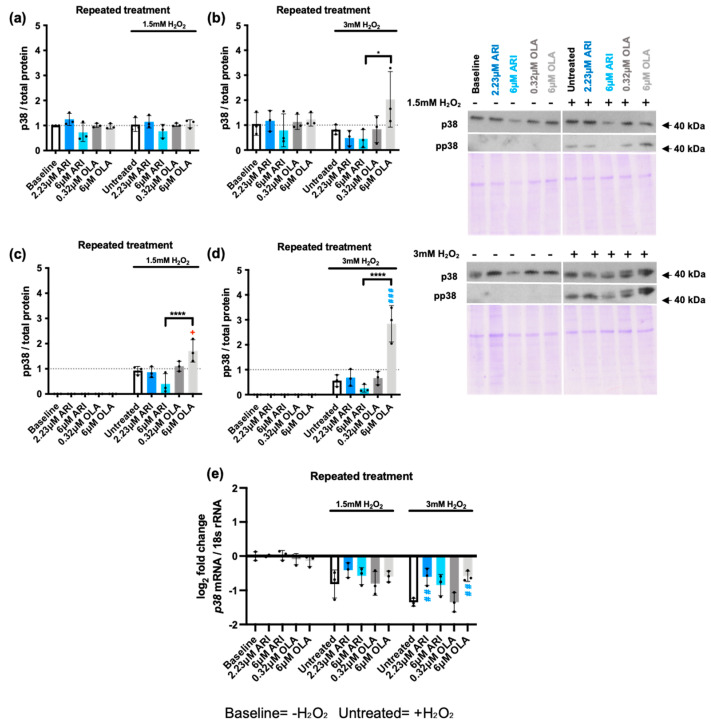
MAPKs: p38. (**a**,**b**) Western blot analysis of total p38 levels in cells challenged with 1.5 mM (**a**) or 3 mM (**b**) H_2_O_2_. (**c**,**d**) Western blot analysis of phospho-p38 (pp38) in cells challenged with 1.5 mM (**c**) or 3 mM (**d**) H_2_O_2_. Panels include graphs and representative Western blots and corresponding Coomassie blue stained gels. (**e**) Logarithmic fold-change in mRNA expression levels of *p38*. Data are presented as mean ± SD and analyzed by one-way ANOVA followed by Dunnett’s test, comparing samples within each group: untreated with treated, without H_2_O_2_ (*), untreated with treated, challenged with 1.5 mM H_2_O_2_ (+); untreated with treated, challenged with 3 mM H_2_O_2_ (#). Additionally, samples are analyzed with Tukey’s test, comparing 6 µM ARI with 6 µM OLA samples (* above the line indicating the comparison). *^/+^ *p* ≤ 0.05, **^##^** *p* ≤ 0.01, **^###^** *p* ≤ 0.001, **** *p* ≤ 0.0001; (**a**–**e**): *n* = 3.

**Figure 5:**
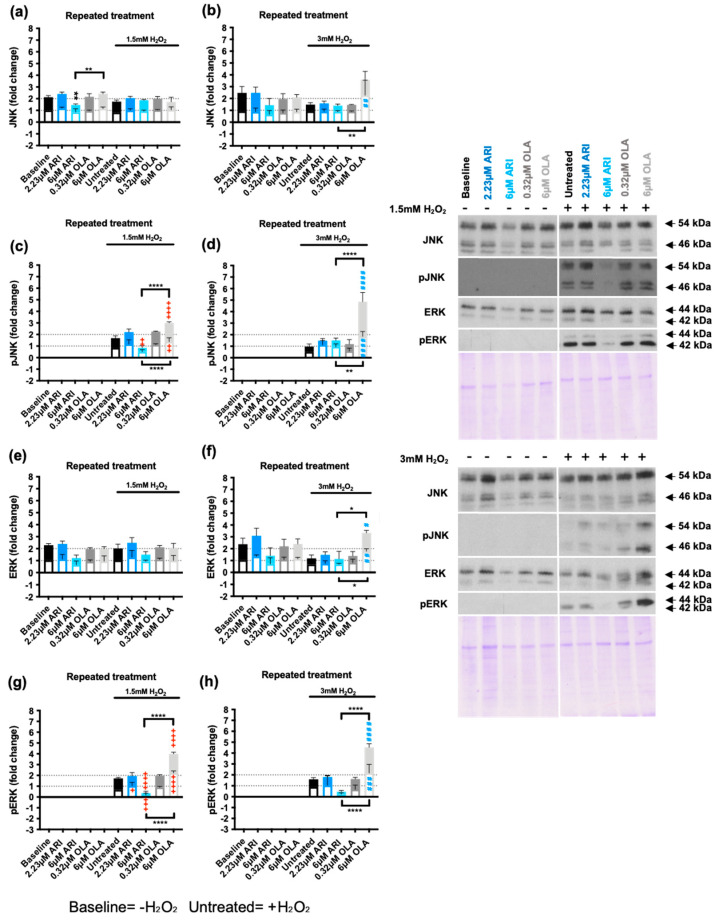
MAPKs: JNK & ERK1/2. (**a**,**b**) Western blot analysis of total JNK levels in cells challenged with 1.5 mM (**a**) or 3 mM (**b**) H_2_O_2_. (**c**,**d**) Western blot analysis of phospho-JNK (pJNK) in cells challenged with 1.5 mM (**c**) or 3 mM (**d**) H_2_O_2_. (**e**,**f**) Western blot analysis of total ERK1/2 levels in cells challenged with 1.5 mM (**e**) or 3 mM (**f**) H_2_O_2_. (**g**,**h**) Western blot analysis of phospho-ERK1/2 (pERK) in cells challenged with 1.5 mM (**g**) or 3 mM (**h**) H_2_O_2_. Panels include graphs and representative Western blots and corresponding Coomassie blue dyed gels. Data are presented as mean ± SD and analyzed by one-way ANOVA followed by Dunnett’s test, comparing samples within each group: untreated with treated, without H_2_O_2_ (*), untreated with treated, challenged with 1.5 mM H_2_O_2_ (+); untreated with treated, challenged with 3 mM H_2_O_2_ (#). Additionally, samples are analyzed with Tukey’s test, comparing 6 µM ARI to 6 µM OLA samples (* above the line indicating the comparison). *^/+/#^ *p* ≤ 0.05, **^/++/##^ *p* ≤ 0.01, ^+++/###^ *p* ≤ 0.001, ****^/++++/####^ *p* ≤ 0.0001; (**a**–**h**): *n* = 3. Legend: filled box represents JNK 46 (**a**,**b**), pJNK 46 (**c**,**d**), ERK 42, i.e., ERK2 (**e**,**f**) and pERK 42 (**g**,**h**). Empty box represents JNK 54 (**a**,**b**), pJNK 54 (**c**,**d**), ERK 44, i.e., ERK1 (**e**,**f**) and pERK 44 (**g**,**h**).

The cellular response to oxidative stress also largely depends on the activation of mitogen activated protein kinases (MAPKs). Indeed, MAPK activity determines whether increased ROS levels drive cell proliferation, activate other stress response nodes, or induce cell death [60]. We therefore evaluated the activation of the three main stress kinases—p38, c-Jun amino-terminal kinases (JNKs), and extracellular signal regulated kinase (ERK1/2)—in response to 1.5 and 3 mM H_2_O_2_ exposure in cells repeatedly treated with ARI/OLA by testing their phosphorylation status. As expected, Western blot analysis showed that both 1.5 and 3 mM H_2_O_2_ exposure significantly increased the phosphorylation of the three MAPKs (Figure 4 and Figure 5). p38 is a multifaceted stress-responsive protein and a recognized ROS- and DNA damage-sensitive protein that induces cell cycle arrest and regulates apoptosis [61]. Total protein levels of p38 were not modified at baseline by the treatments (Figure 4a,b) or by H_2_O_2_ exposure, with the exception of cells treated with 6 μM OLA and exposed to 3 mM H_2_O_2_. Regarding p38 activation, cells treated with 6 μM OLA and 1.5/3 mM H_2_O_2_ also had significantly higher p38 phosphorylation levels than untreated cells exposed to H_2_O_2_ (Figure 4c,d). Furthermore, in cells exposed to both 1.5 and 3 mM H_2_O_2,_ which increased p38 phosphorylation in untreated cells, there was a significant difference in p38 phosphorylation between 6 μM OLA- and 6 μM ARI treated cells, with the former inducing higher p38 phosphorylation levels (Figure 4c,d). We next evaluated *p38* gene expression levels. Results showed that at baseline (without H_2_O_2_), the treatments did not modify p38 expression (Figure 4e), whereas 1.5 and 3 mM H_2_O_2_ exposure induced a significant downregulation of gene expression, and this was partially prevented by ARI/OLA treatments. The response to 1.5 mM H_2_O_2_ was not significantly modified by the treatments, but in cells challenged with 3 mM H_2_O_2_, both 2.23 μM ARI and 6 μM OLA treatments maintained significantly higher levels of *p38* mRNA than those of untreated controls (Figure 4e). Accordingly, neither the observed hypersensitivity to p38 phosphorylation induced by OLA nor the reduced sensitivity induced by ARI could be attributable to gene expression changes, as both treatments seem to promote the maintenance of *p38* levels. Notably, this gene expression pattern is remarkably similar to that previously observed for pro- and anti-apoptotic factors with the exception of *Mcl1*.

We next evaluated JNK, a MAPK that can promote both cell survival and apoptosis in response to oxidative stress [62], and also ERK1/2, which has recognized proliferative and protective effects on cells exposed to oxidative stress [63]. Under baseline conditions, JNK levels were significantly lower in 6 μM ARI-treated cells than in untreated cells, whereas levels after 6 μM OLA treatment were similar to those of untreated cells (Figure 5a,b). By contrast, JNK levels were increased following the 3 mM H_2_O_2_ challenge in 6 μM OLA treated cells, and under these conditions, the difference between the 6 μM ARI and 6 μM OLA groups was significant. Consistently, JNK phosphorylation in response to 1.5 mM H_2_O_2_ was significantly decreased in cells treated with 6 μM ARI and increased in cells treated with 6 μM OLA, with differences between the two being significant (Figure 5c). Furthermore, JNK phosphorylation in response to 3 mM H_2_O_2_ was increased in 6 μM OLA-treated cells and the difference between 6 μM ARI- and 6 μM OLA-treated cells was also significant (Figure 5d). Therefore, the effects of ARI and OLA on p38 and JNK are consistent with ARI reducing and OLA enhancing the response to H_2_O_2_.

Similar, although ERK1/2 levels were not significantly modified by any of the treatments under baseline conditions (Figure 5e,f), in cells challenged with 3 mM H_2_O_2_, ERK1/2 levels were significantly higher in 6 μM OLA-treated cells than in untreated controls and than in 6 μM ARI treated cells. Also, the level of ERK1/2 phosphorylation in response to 1.5 mM H_2_O_2_ was significantly lower in 6 μM ARI-treated cells than in untreated controls, whereas the levels of ERK1/2 phosphorylation in response to 1.5/3 mM H_2_O_2_ where significantly higher in 6 μM OLA-treated cells than in untreated controls or in cells treated with 6 μM ARI (Figure 5g,h). In summary, while treatment with 6 μM ARI reduced JNK and ERK activation in response to an oxidative challenge, 6 μM OLA treatment increased p38, JNK, and ERK1/2 activation in response to H_2_O_2_. This suggests that while ARI tends to reduce MAPK responsiveness to H_2_O_2_ exposure, OLA treatment has the opposite effect. These findings are consistent with the changes observed in pro- and anti-apoptotic factors. These effects may be related to the basal oxidative stress levels experienced by the cells, as ROS can alter the activity of regulatory phosphatases, peroxiredoxins, and tyrosine kinases, reducing the sensitivity of signal transduction systems [63].

**Figure 6:**
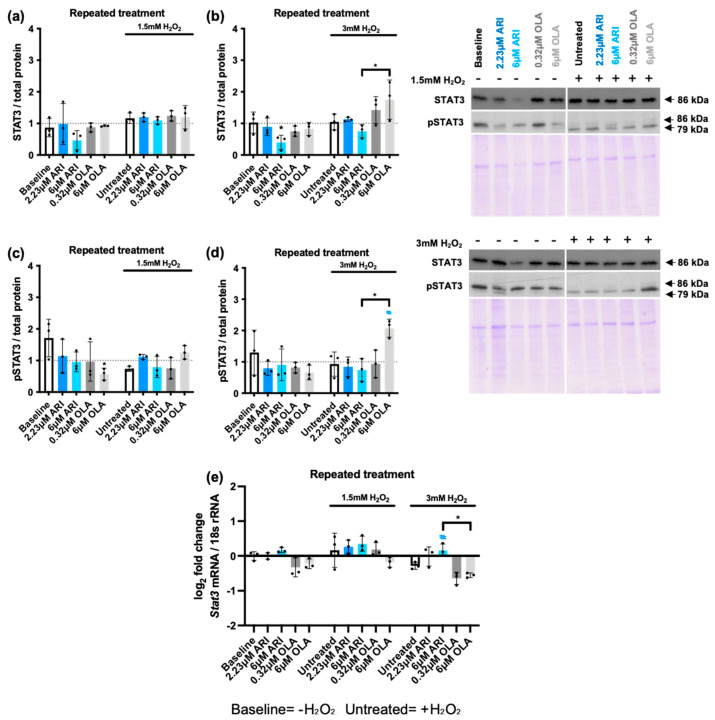
STAT3. (**a**,**b**) Western blot analysis of total STAT3 levels in cells challenged with 1.5 mM (**a**) or 3 mM (**b**) H_2_O_2_. (**c**,**d**) Western blot analysis of phospho-STAT3 (pSTAT3) in cells challenged with 1.5 mM (**c**) or 3 mM (**d**) H_2_O_2_. Panels include graphs and representative Western blots and corresponding Coomassie blue dyed gels. (**e**) Logarithmic fold-change in mRNA expression levels of *Stat3*. Data are presented as mean ± SD and analyzed by one-way ANOVA followed by Dunnett’s test, comparing samples within each group: untreated with treated, without H_2_O_2_ (*), untreated with treated, challenged with 1.5 mM H_2_O_2_; untreated with treated, challenged with 3 mM H_2_O_2_ (#). Additionally, samples were analyzed with Tukey’s test, comparing 6 µM ARI to 6 µM OLA samples (* above the line indicating the comparison). *^/#^ *p* ≤ 0.05; (**a**–**e**): *n* = 3.

Another critical regulator of the cellular stress response is the transcription factor STAT3 [64], which generally exerts a cytoprotective function by preventing cellular apoptosis [65]. Because the interplay between MAPKs and STAT3 signaling pathways is pivotal during the cellular response to oxidative stress [66], we examined STAT3 levels in repeatedly treated cells and found a reduction at baseline in cells treated with 6 μM ARI. In cells challenged with 3 mM H_2_O_2_, the levels of STAT3 were significantly higher in 6 μM OLA-treated cells than in 6 μM ARI-treated cells (Figure 6b). We then evaluated STAT3 phosphorylation as a surrogate marker of its activation status. Consistently, we found that STAT3 phosphorylation following challenge with 3 mM H_2_O_2_ was higher in 6 μM OLA-treated cells than in 6 μM ARI-treated cells (Figure 6d). Analysis of *Stat3* gene expression revealed that at baseline conditions, the treatments did not modify *Stat3* levels. Following exposure to 3 mM H_2_O_2_, when *Stat3* levels were reduced in untreated cells, the treatment with 6 μM OLA resulted in a significantly larger downregulation than in the cells treated with 6 μM ARI. These results, along with the observed changes in MAPK activity and pro- and anti- apoptotic factor regulation, suggest that overall, ARI reduces and OLA enhances stress sensitivity, and these changes in responsiveness affect pro-survival and pro-apoptotic pathways. Notably, while the ARI-dependent reduced sensitivity might be related to the observed oxidative stress known to reduce the sensitivity of many signaling pathways, and in particular Tyr-receptor signaling, the mechanism involved in OLA-dependent enhanced sensitivity needs clarification.

**Figure 7:**
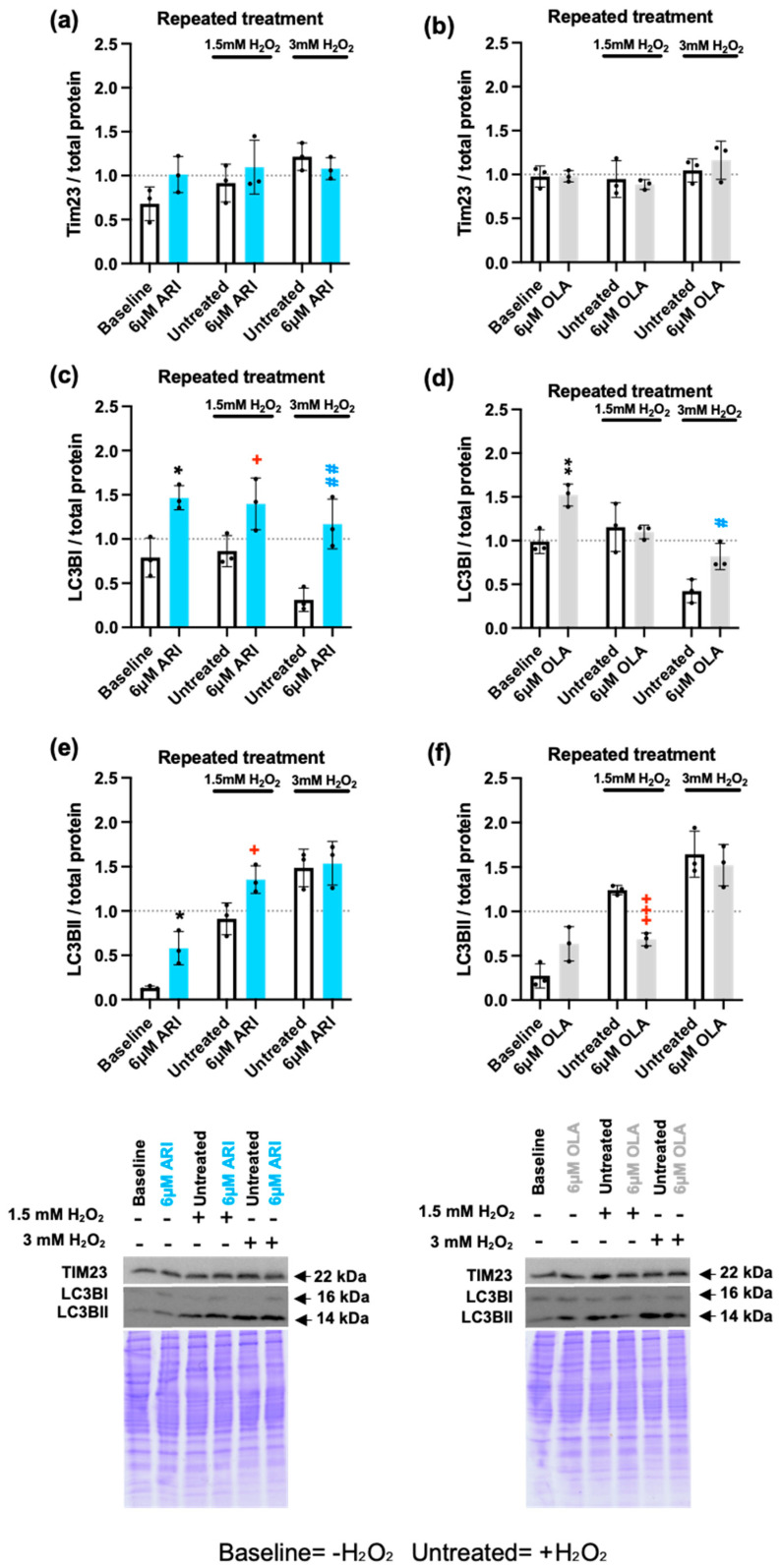
Mitochondria load and Autophagy. (**a**,**b**) Western blot analysis of Tim23 in cells treated with ARI (**a**) or OLA (**b**) and then challenged with 1.5/3 mM H_2_O_2_. (**c**,**d**) Western blot analysis of LC3BI in cells treated with ARI (**c**) or OLA (**d**) and then challenged with 1.5/3 mM H_2_O_2_. (**e**,**f**) Western blot analysis of LC3BII in cells treated with ARI (**e**) or OLA (**f**) and then challenged with 1.5/3 mM H_2_O_2_. Panels include graphs and representative Western blots and corresponding Coomassie blue dyed gels. Data are presented as mean ± SD and analyzed with the Unpaired *t* test, comparing samples within each group: untreated with treated, without H_2_O_2_ (*), untreated with treated, challenged with 1.5 mM H_2_O_2_ (+); untreated with treated, challenged with 3 mM H_2_O_2_ (#).*^/+/#^ *p* ≤ 0.05, **^/##^ *p* ≤ 0.01, ^+++^ *p* ≤ 0.001; (**a**–**f**): *n* = 3.

As alterations in mitochondrial function seemed to be the primary driver in the differential response to H_2_O_2_, we next determined whether mitochondrial load, or mitochondrial turnover, were modified by repeated ARI or OLA treatments. Oxidative stress-damaged mitochondria are targeted for mitophagy, a specialized form of autophagy, and mitochondrial biogenesis is activated to compensate for this loss [23] to maintain the cellular energy balance. Total mitochondria content was determined by Western blotting with an antibody directed against the mitochondrial protein Tim23, part of the TIM23 complex localized in the IMM, which drives mitochondrial protein import into the IMM and the mitochondrial matrix [67]. No differences were found in Tim23 amounts between the groups either at baseline or in response to 1.5/3 mM H_2_O_2_ (Figure 7a,b). Detection of mitophagy by this method was confirmed in conditions of starvation with the observation of reduced Tim23 levels (Supplementary Figure S1a). We next evaluated autophagy using Western blot analysis of microtubule-associated protein 1A/1B-light chain 3 (LC3B). The cytosolic form of LC3B (LC3BI) is recruited to phagosome membranes following conjugation with phosphatidylethanolamine, forming LC3BII. Therefore, the LC3BI/II ratio is commonly used as an indicator of autophagy flux. We found that cells treated with 6 μM OLA, both at baseline and following challenge with 3 mM H_2_O_2_, had significantly higher levels of LC3BI than the corresponding untreated controls, whereas ARI significantly modified LC3BI levels in all of the conditions tested (Figure 7c,d). In challenge experiments with 1.5 mM H_2_O_2_, cells treated with 6 μM ARI had significantly higher levels of LC3BII, while cells treated with 6 μM OLA had significantly lower LC3BII levels than untreated controls (Figure 7e,f). These results suggest that ARI-treated cells may have enhanced autophagy flux, likely to remove damaged mitochondria exposed to high levels of O_2_^•-^, whereas OLA treatment leads to a reduction in autophagy levels.

**Figure 8:**
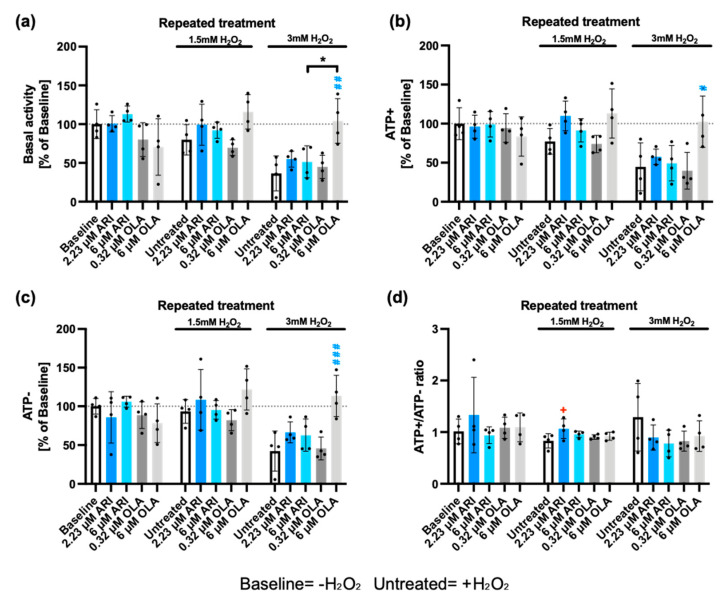
Proteasome activity. (**a**) Basal conditions; (**b**) with added ATP, ATP+; (**c**) following ATP depletion, ATP−; (**d**) ratio of ATP+ and ATP− activity determinations. Data are presented as mean ± SD and analyzed with one-way ANOVA followed by Dunnett’s test, comparing samples within each group: untreated with treated, without H_2_O_2_ (*), untreated with treated, challenged with 1.5 mM H_2_O_2_ (+); untreated with treated, challenged with 3 mM H_2_O_2_ (#). Additionally, samples are analyzed with Tukey’s test, comparing 6 µM ARI to 6 µM OLA samples (* above the line indicating the comparison). *^/+/#^ *p* ≤ 0.05, **^##^** *p* ≤ 0.01, **^###^** *p* ≤ 0.001; (**a**–**d**): *n* = 4.

Elevated ROS levels increase the cellular burden of oxidized/damaged proteins, which are typically targeted for degradation by the proteasome. However, both ROS themselves and the reduction in mitochondrial oxidative capacity can negatively impact proteasome activity, leading to the accumulation of damaged proteins. This accumulation can ultimately contribute to cellular dysfunction and even cell death [68,69]. There are two main proteasome complexes: the 26S complex requires ATP and targets ubiquitinated proteins; it is also responsible for ≥80% of the total proteasome activity [70]. The remaining 20% is degraded by the 20S proteasome complex, which is both ubiquitin- and ATP-independent [71]. Both 26S and 20S proteasome complexes can degrade oxidatively damaged proteins, but the 20S complex is the main protease responsible for this, likely because it is more resistant to ROS [72]. Therefore, we examined whether repeated treatments with ARI and OLA modified the proteasomal response of cells exposed to 1.5/3 mM H_2_O_2_. Basal proteasomal activity, following the addition (ATP+) and depletion (ATP−) of ATP, was determined as well as the ATP+/ATP− activity ratio. As previously reported [73], 3 mM H_2_O_2_ reduced both 26S (ATP-dependent) and 20S (ATP-independent) proteasome activity (Figure 8). Notably, neither depletion nor addition of ATP significantly modified the inhibition rate, and even though the challenge with 3 mM H_2_O_2_ produced a greater inhibition of the ATP-dependent activity, differences for the ATP+/ATP− activity ratio were not significant (Figure 8d).

ARI treatment did not significantly alter the ATP-dependent (26S) (Figure 8b) or ATP-independent (20S) proteasome activities compared with baseline (Figure 8c). However, 2.23 μM ARI significantly increased the ATP+/ATP− activity ratio (Figure 8d), suggesting better maintenance of 26S activity than 20S activity. Notably, cells treated with 6 μM OLA and challenged with 3 mM H_2_O_2_ showed significantly higher proteasomal activity than untreated cells, recovering the proteasomal activity levels observed in the absence of H_2_O_2_ (Figure 8b,c). These observations imply that OLA treatment has an important general protective role on the ROS-dependent inactivation of the proteasome by H_2_O_2_, whereas ARI treatment has a small but significant effect on the maintenance of the 26S proteasome. This could be related to the higher levels of antioxidants over baseline. The protection elicited by OLA is likely more related to the lower load of damaged mitochondria [74] than to the higher sensitivity to MAPK activation, as p38 has been shown to inhibit proteasome activity [75].

In summary, ARI-treated cells produce higher levels of mitochondrial ROS and induce stronger mitophagy and mitochondria-dependent apoptosis in responses to H_2_O_2_ exposure. However, these cells are more resistant to death than control cells, possibly because the stress signaling pathways are blunted. This is supported by the reduced activation of the MAPKs and STAT3 under stress, as well as dampened gene expression changes in pro- and anti-apoptotic factors in response to ROS. By contrast, OLA treatment reduces the mitophagy flux, hyper-sensitizes cells to stress signaling, and protects proteasomal activity in cells exposed to high ROS levels, suggesting it has a sensitizing effect on cell death in stress conditions despite the lower cellular damage.

## 3. Discussion

The potential hazards derived from the mitochondrial toxicity of pharmacological drugs are a growing concern, especially for patients undergoing lifelong treatment [76]. Neuropharmacology poses particular challenges due to the need for drugs to cross the Blood–Brain Barrier (BBB). Additionally, circulating drug concentrations must be relatively high to ensure therapeutic levels cross the BBB [77]. This likely contributes to the observed increased incidence of metabolic diseases that can be attributable to pharmacological treatments in patients [78]. Under the umbrella of mitochondrial toxicity or mitochondrial dysfunction, a diverse range of possible biological effects can arise from the interaction of pharmacological drugs with normal mitochondrial physiology. The multifaceted nature of these pathological outcomes is exemplified by the often perplexing phenotypic variability observed in mitochondrial genetic diseases [79]. Notably, to date, there are no established protocols that can accurately assess these conditions or reliable risk assessments for disease development [80].

Here, we aimed to understand how two widely used AAPs, ARI and OLA, which have been demonstrated to inhibit mitochondrial oxidative phosphorylation, modify cellular physiology by altering the capacity to respond to stress. This concept is grounded in previous research indicating a strong correlation between mitochondrial oxidative function and the regulation of cellular stress response mechanisms, ultimately providing an effective mechanism against diverse pathological conditions [25,81].

Our study demonstrates that treatment with ARI, but not with OLA, leads to increased levels of oxidative stress in mitochondria. This observation is consistent with a recent publication demonstrating that ARI is a respiratory chain Complex I inhibitor [46]. This increased stress is a probable driver of enhanced mitophagy flux in ARI-treated cells and is associated with a consistently higher sensitivity to mitochondria-driven apoptosis. While oxidative stress is known to induce apoptosis in a wide variety of cell types, ARI-treated cells exhibit reduced sensitivity to typical stress-induced changes. This lack of sensitivity encompasses a loss of transcriptional regulation of genes related to apoptosis control and reduced activation of both MAPKs and STAT3. Conversely, OLA treatment has the opposite effect. These observations are consistent with the known effect of persistent cellular oxidative stress, which can inactivate key signaling mediators such as membrane receptors and regulatory phosphatases. Consequently, the observed reduced cell death rates in ARI-treated cells are likely attributable to this lack of responsiveness. How and if ARI and OLA impact alternative cell-death pathways needs to be established. Of particular interest in the liver is the ferroptosis pathway, since it is closely related to elevated ROS levels [57].

In contrast, OLA treatment induced hypersensitivity to stress responses at all tested levels (gene expression of apoptosis regulators, MAPK and STAT3 activation, proteasome activity). This heightened sensitivity cannot be attributable to detectable differences in mitochondrial oxidative stress, as detected by MitoSOX labeling of O_2_^•-^, to global changes in oxidative stress that affect the 26S/20S activity ratio, or to changes in mitochondria levels, evaluated through the determination of Tim23 protein levels. Notably, OLA appeared to reduce autophagy in H_2_O_2_-treated cells, or perhaps, more accurately, the induction of autophagy by H_2_O_2_ was reduced in OLA-treated cells. While this result may seem counterintuitive given the similar antioxidant activity between OLA-treated cells and control cells [56], it could be explained by the increased activity of the proteasome detected in H_2_O_2_-challenged OLA-treated cells, which would likely increase the cellular capacity to remove oxidatively damaged proteins.

The elevated proteasome activity observed in OLA-treated cells may contribute to their heightened sensitivity to MAPK activation. This relationship is particularly well established for ERK, whose activation in response to oxidative stress has been shown to be proteasome dependent [82]. Proteosome inactivation prevents the activation of the upstream kinase MEK, blocking ERK activation [83].

While oxidative stress is a well-defined activator of the proteasome, chronic exposure to ROS and mitochondrial dysfunction tend to reduce proteasome activity. The critical role of the proteasome in ROS-dependent effects is highlighted by its role in the activation of the inflammasome, a process that is also dependent on MAPK activation [84].

The elevated proteasome activity observed in OLA-treated cells exposed to H_2_O_2_ was not accompanied by any detectable differences in baseline proteasome levels or by significant changes in baseline MAPK levels or activity. Consequently, the origin of this conditioning effect remains to be established. However, a notable exception in OLA-treated cells was a significant increase in LC3BI levels without a corresponding reduction in LCBII. This suggests that the increase in LC3BI is not indicative of autophagy flux inhibition but rather reflects an enhanced capacity of the cells to induce autophagy and, subsequently, to degrade damaged proteins/structures. Although highly speculative, this finding may be linked to the observed sensitivity of proteasome activation in response to OLA. Moreover, given the differential effects of ARI and OLA, it is tempting to speculate that mitochondrial function may play a role.

Total LC3 levels are regulated by acetylation, a process that prevents their degradation by the proteasome [85]. Under fasting conditions, increased NAD^+^/NADH ratios activate the deacetylase SIRT1 [86], rendering the protein more sensitive to proteasomal degradation while simultaneously activating mitophagy flux, as it facilitates the conjugation of phosphatidylethanolamine to pre-autophagic membranes [87]. Therefore, elevated levels of LC3B can be indicative of a good cellular nutritional and redox status. All these possibilities need to be further investigated.

Our observations elucidate the differential impact of drug-induced mitochondrial toxicity on physiological responses to stress. These findings highlight the critical role played by chronic mitochondrial oxidative stress exposure in cellular stress desensitization and raise a note of caution on the potential pathological outcomes of long-term ARI treatment. In clinical practice, the observation that ARI attenuates the response to stress signals could be related, for example, to an increased risk of developing hepatocarcinoma. Failure to respond to stress signals can lead to cell transformation. It is therefore recommended to assess mitochondrial health before prescribing medication and during patient follow-up to minimize secondary risks.

## 4. Materials and Methods

### 4.1. Reagents

Reagents were purchased from Sigma-Aldrich (Merck, Darmstadt, Germany), unless otherwise indicated.

### 4.2. Cell Culture

The rat hepatoma cell line Fao (#89042701, ECACC) was grown at 37 °C in a humidified atmosphere with 5% CO_2_, in Coon’s F-12 Modified Liquid Medium (#MBC-F0855) supplemented with 10% fetal bovine serum (#10270-106)), 1% penicillin/streptomycin (#15140-122) and 1% GlutaMAX supplement (#35050038) (all from Gibco, Thermo Fisher Scientific, Waltham, MA, USA). Cells were seeded on 96-well flat-bottom culture plates (40,000 cells/well) for cytotoxicity assessments, collagen-coated (5 μg/cm^2^) 1 cm^2^ glass cover slips on 24-well plates (200,000 cells/well) for MitoSOX assessment, 12-well culture plates (500,000 cells/well) for Caspase activity determination, 6-well culture plates (1 million cells/well) for proteasomal activity measurements, and 6-well culture plates (1 million cells/well) and T25 flasks (3 million cells/flask) for gene and protein expression analyses, respectively. To test the effects of ARI and OLA, Fao cells were grown in the presence of vehicle (0.12% DMSO; #67-68-5, Acros Organics, Thermo Fisher Scientific), ARI (#PHR1784), or OLA (#PHR1825) at doses corresponding to laboratory alert levels in patient sera (2.23 μM ARI and 0.32 μM OLA) or at 6 μM ARI/OLA, a supraphysiological concentration for plasma levels that aims to mimic the drug’s accumulation in the liver, which has been previously tested in vitro [38] and stays soluble in culture media. Different batches of cells were tested in parallel. Cells were exposed to ARI/OLA for 24 h (single treatment) or 4–8 weeks (repeated treatment) with media changes every 24 h prior to a challenge with 1.5 or 3 mM H_2_O_2_ for 3 h, followed by harvesting. Biological replicate values were divided by the average of all samples in the replicate to account for multi-plate experiment variability, and expressed normalized to the baseline sample. For a detailed description of the repeated treatments, please refer to [56]. A minority of data in Figure 1 and Figure 2, those of H_2_O_2_-untreated experiments, has been published previously to cover different aspects of the study (described in the legends to Figure 1 and Figure 2). All data (biological replicates) presented were simultaneously recorded and are complete biological replicates from the same experimental setup.

### 4.3. MitoSOX Cells

MitoSOX Cells were seeded on collagen-coated glass coverslips 24 h prior to treatment. Following treatment, cells were washed with PBS and labeled with 3 μM MitoSOX in HBSS (1.26 mM CaCl_2_, 0.49 mM MgCl_2_ × 6H_2_O, 0.41 mM MgSO_4_ × 7H_2_O, 5.33 mM KCl, 0.44 mM KH_2_PO_4_, 4.17 mM NaHCO_3_, 137.93 mM NaCl, 0.34 mM Na_2_HPO_4_, 5.56 mM D-glucose) in the dark for 10 min at 37 °C. Cells were then washed with PBS, fixed with 4% paraformaldehyde for 15 min at room temperature, washed again with PBS, and stored at 4 °C in the dark prior to mounting on slides with ProLong Diamond Antifade Mountant (Thermo Fisher Scientific). Images were captured using an inverted fluorescence microscope (Olympus IX81F, Olympus Corporation, Tokyo, Japan). The scale bar for images is 50 μm. The fluorescent signals were divided by the average signal of all samples in the biological replicate, then normalized to the signal of baseline.

### 4.4. Cell Viability

Cytotoxicity was evaluated using 0.04 mg/mL NR (3-Amino-7-dimethylamino-2-methylphenazine hydrochloride; N4638) staining, as described in [38]. The NR assay was performed for single ARI/OLA treatments. Dehydrogenase activity was determined using the Thiazolyl Blue Tetrazolium Bromide assay (MTT, #M5655). The extracellular release of LDH was measured using the LDH Cytotoxicity Detection Kit (#MK401, Takara Bio Europe, Saint-Germain-en-Laye, France), as described [56]. Images were captured using an inverted microscope (Leica, Wetzlar, Germany). Representative images are included in Supplementary Figure S2. The scale bar for images is 50 μm.

### 4.5. Caspase Activity

Caspase 3/7 and 9 activities were assessed using Caspase-Glo reagents (#G810C, #G811C #G816C, #G822C, Promega, Madison, WI, USA). Caspase cleavage releases an active luciferase substrate whose luminescence signal is proportional to Caspase activity. This assay was used following ARI and OLA treatments with and without the 3 h H_2_O_2_ challenge, as well as with cells treated for 3 h with 1 μM STS, prior to harvesting. Cells were washed with PBS and stored at −80 °C. Cells were then lysed in CCLR lysis buffer (#E1531, Promega), centrifuged, and the supernatant was saved, diluted with CCLR, and Caspase-Glo 9 or Caspase-Glo 3/7 reagent was added. Following a 20 min incubation, the luminescence signal was detected with a luminometer 20/20n (Turner Biosystems Corp., Promega). The protein concentration of the extract was determined using the Pierce 660 nm Protein Assay Reagent (Thermo Fisher Scientific). It was used to determine the luminescence/μg of protein in the sample.

### 4.6. mRNA Analysis

Total RNA was isolated and reverse-transcribed as described [38]. Gene expression was assessed by qPCR analysis using TaqMan probes on a 7500 Real-Time PCR System using SDS v1.3.1 software (Applied Biosystems, Thermo Fisher Scientific). Gene expression was calculated relative to the expression of the reference gene, 18S rRNA (#Rn18s), using the following equation: (1 + E_reference_)^Ct,reference^/(1 + E_target_)^Ct,target^, where E is the PCR efficiency and Ct is the threshold cycle [88,89,90], as described [38], and is expressed as logarithmic fold change normalized to the baseline sample. The following TaqMan probes labeled with FAM dye (Thermo Fisher Scientific) were used: the reference gene Rn18s (#Rn03928990_g1), *Cas3* (#Rn00563902_m1), *Cas9* (#Rn00581212_m1), *p38* (#Rn00578842_m1), *Stat3* (#Rn00680715_m1), *Bax* (#Rn01509178_m1), *Bcl2* (#Rn99999125_m1), *BclXL* (#Rn00437783_m1), *Mcl1* (#Rn00821024_g1), *Bid* (#Rn01459517_m1), and *Diablo* (#Rn01480487_g1).

### 4.7. Protein Extraction and Western Blotting

Whole-cell lysates were obtained as described [91]. In brief, 20 μg of whole protein extract was loaded onto 10% and 12% acrylamide gels for separation by standard sodium dodecyl sulfate-polyacrylamide gel electrophoresis (SDS-PAGE). We used in-house cast gels prepared with 40% Acrylamide/Bis Solution, 37.5:1 (Bio-Rad, Hercules, CA, USA, #1610148) containing a 5% stacking gel. The gel was stained with Coomassie blue (#27813, Fluka, Milwaukee, WI, USA) to control for equal loading, and proteins were transferred onto PVDF Immobilon-P membranes (#IPVH00010) using a semi-dry transfer system (BioRad, Hercules, CA, USA). Membranes were blocked with 3% BSA in TBS-T and incubated overnight at 4 °C with primary antibodies, washed with TBS-T, and then incubated with horseradish peroxidase-conjugated goat anti-rabbit or goat anti-mouse secondary antibodies (#1706515 and #1706516, respectively, BioRad), washed again and developed with Clarity ECL Substrate (BioRad). The luminescence signal was captured on X-ray films (AGFA, CP-BU, 0413), developed on a Curix60, 9462 developing machine, and then quantified using Image Studio software v.5.2.5. (LI-COR, Lincoln, NE, USA). Experimental replicates on separate membranes were normalized by calculating the ratio of individual values by the average signal for all samples in the membrane. Primary antibodies used were from Cell Signaling Technology (Danvers, MA, USA): Phospho-p38 MAPK (Thr180/Tyr182) (#9211), p38 MAPK (#9212), Phospho-SAPK/JNK (Thr183/Tyr185) (#9251), SAPK/JNK (#9252), Phospho-p44/42 MAPK (Erk1/2) (Thr202/Tyr204) (#9101), p44/42 MAPK (Erk1/2) (#9102), Phospho-Stat3 (Tyr705) (D3A7) XP Rabbit mAb (#9145), Stat3 (D3Z2G), Rabbit mAb (#12640); from Abcam (Cambridge, UK): Bcl-xL (#2762), LC3B (#2775); and from BD transduction Laboratories (Franklin Lakes, NJ, USA): Tim23 (#611222). Raw data are included in Supplementary Figures S1, S3 and S4.

### 4.8. Proteasome Activity 

Proteasome activity was determined as described [92] using a procedure based on 7-Methoxycoumarin-4-acetic acid (MCA) fluorescence, which is released following cleavage of the fluorogenic peptide suc-LLVY-MCA in the presence of chymotrypsin-like proteasome activity. The method allows the separate determination of 20S and the 26S proteasome activities by using their differential requirement for ATP. The 20S subunit does not require ATP, while the proteolytic activity of the fully assembled 26S proteasome does. In brief, following treatments, cell culture plates were washed with PBS and stored at −80 °C. After thawing, cells were scraped with a rubber spatula in proteasome assay (PA) lysis buffer (250 mM sucrose, 30 mM HEPES, 20 mM MgCl_2_, 1.3 mM EDTA, 1.6 mM DTT, pH 7.8), homogenized using a 0.5 mm sonotrode (UP50H, Hielscher, Germany), in sets of three, with 10 × 0.5-s pulses at 80% amplitude, and then centrifuged. The supernatants were stored at −80 °C. Following thawing, 10 μL of the sample was placed in black 96-well plates along with the reaction mixture corresponding to the proteasome to be analyzed (20S: 1.8 μM DTT, 16 mM deoxyglucose, 0.1 mg/mL hexokinase in PA; 26S: 1.8 μM DTT 4.5 mM ATP in PA). The reaction was activated by the addition of 180 μM suc-LLVY-MCA substrate. After 1 h incubation at 37 °C, the fluorescence was measured in a Victor3 reader, excitation 355 nm, and emission 460 nm (Perkin Elmer, Waltham, MA, USA). Proteasome activity was estimated as the amount of MCA released per time unit per μg of protein, predetermined with the Pierce 660 nm Protein Assay Reagent (Thermo Fisher Scientific).

### 4.9. Statistical Analysis

Statistical analysis was performed with GraphPad Prism 10.1.1 using its inbuilt algorithm to test the equality of variances from medians with the Brown–Forsythe test. One-way ANOVA was used to compare the effect of drug treatments in case of equal variances, followed by Dunnett’s multiple-comparison test (when testing multiple experimental groups against a single control group) or Tukey’s multiple-comparison test (when studying the relationship between variables). The Unpaired *t* test was used when comparing two samples (LC3B data). Two-way ANOVA was used on two sets of data to examine how two independent variables, in combination, affect a dependent variable. This was used to test for the interaction of the phosphorylated and non-phosphorylated forms of the proteins JNK and ERK derived from Western blot analysis. Data from statistical tests (Brown–Forsythe, One- and Two-way ANOVA, *t*-test) were compiled and are included in Supplementary Tables S1–S5. Treated samples were compared with the baseline and untreated controls. All data are presented as mean ± standard deviation (SD), and the significance level was set at *p* < 0.05. Statistical parameters, sample sizes (*n*), and *p*-values are noted in the figure legends.

## Data Availability

Data are available as part of the Supplementary Materials.

## Supplementary Materials

The following supporting information can be downloaded at: https://www.mdpi.com/article/10.3390/ijms252011119/s1.
