# Supplementary material for: Aripiprazole, but Not Olanzapine, Alters the Response to Oxidative Stress in Fao Cells by Reducing the Activation of Mitogen-Activated Protein Kinases (MAPKs) and Promoting Cell Survival
Source: Int J Mol Sci. 2024 Oct 16;25(20):11119. doi: 10.3390/ijms252011119 (PMC11508229; doi:10.3390/ijms252011119)
Supplement: Supplementary file 1 [file ijms-25-11119-s001.zip › Supplementary tables-20240818.pdf]

# Supplementary Table S1

Compared to Baseline

| | Brown-Forsythe test | | One-way ANOVA | | 6 μM ARI *vs* 6 μM OLA |
|---|---|---|---|---|---|
| Fig | F | *p*-value | F | *p*-value | *p*-value |
| 1a | 0.3568 | 0.8711 | **17.67** | **<0.0001** | **<0.0001** |
| 1b | 0.2965 | 0.9903 | **21.41** | **<0.0001** | n.a. |
| 1c | 0.3729 | 0.9777 | **46.50** | **<0.0001** | n.a. |
| 1d | 1.261 | 0.2687 | **76.81** | **<0.0001** | n.a. |
| 1e | 1.070 | 0.402 | **72.21** | **<0.0001** | n.a. |
| 1f | 1.485 | 0.149 | **30.89** | **<0.0001** | n.a. |
| 1g | **2.373** | **0.0087** | **11.92** | **<0.0001** | n.a. |
| 2a | 0.4844 | 0.9297 | **16.29** | **<0.0001** | n.a. |
| 2b | 0.9960 | 0.4732 | **67.02** | **<0.0001** | n.a. |
| 2c | 1.493 | 0.1529 | **10.27** | **<0.0001** | n.a. |
| 2d | 0.5270 | 0.9037 | **10.2** | **<0.0001** | n.a. |
| 2g | 0.6099 | 0.8355 | **5.881** | **<0.0001** | n.a. |
| 2h | 0.5258 | 0.8981 | **2.872** | **0.0075** | n.a. |
| 3a | 0.2622 | 0.9947 | 7.971 | **<0.0001** | n.a. |
| 3b | 0.4650 | 0.881 | 9.866 | **<0.0001** | **0.0286** |
| 3c | 0.3725 | 0.973 | **4.839** | **0.0001** | n.a. |
| 3d | 0.8681 | 0.5962 | **14.06** | **<0.0001** | n.a. |
| 3e | 0.5595 | 0.8746 | **6.356** | **<0.0001** | n.a. |
| 3f | 1.400 | 0.1925 | **3.072** | **0.0022** | n.a. |
| 3g | 0.4904 | 0.9203 | **8.472** | **<0.0001** | n.a. |
| 4a | 0.5479 | 0.8224 | 1.574 | 0.1902 | n.a. |
| 4b | 0.5480 | 0.8223 | 2209 | 0.0672 | n.a. |
| 4c | n.a. | n.a. | n.a. | n.a. | n.a. |
| 4d | n.a. | n.a. | n.a. | n.a. | n.a. |
| 4e | 0.5188 | 0.9028 | **11.66** | **<0.0001** | n.a. |
| 6a | 0.9561 | 0.5019 | 1.964 | 0.1001 | n.a. |
| 6b | 0.5819 | 0.7964 | **4.358** | **0.0029** | n.a. |
| 6c | 0.5536 | 0.8181 | 2.226 | 0.0654 | n.a. |
| 6d | 0.4012 | 0.9198 | **3.3** | **0.0125** | n.a. |
| 6e | 0.5221 | 0.9006 | **5.96** | **<0.0001** | n.a. |
| 8a | 1.161 | 0.3364 | **6.222** | **<0.0001** | n.a. |
| 8b | 0.7010 | 0.7614 | **4.98** | **<0.0001** | n.a. |
| 8c | 0.9221 | 0.543 | **4.663** | **<0.0001** | n.a. |
| 8d | **2.113** | **0.0294** | 1.022 | 0.4497 | n.a. |

Compared to Untreated control with 1.5 mM $H_2O_2$

| | Brown-Forsythe test | | One-way ANOVA | | 6 μM ARI *vs* 6 μM OLA |
|---|---|---|---|---|---|
| Fig | F | *p*-value | F | *p*-value | *p*-value |
| 1a | 0.1927 | 0.8280 | **4.562** | **0.0429** | **0.0006** |
| 1b | 0.06782 | 0.9903 | 0.5430 | 0.7082 | n.a. |
| 1c | 0.4211 | 7915 | **9.198** | **0.0002** | **0.0001** |
| 1d | 0.7226 | 0.5899 | 0.2401 | 0.9112 | n.a. |
| 1e | 0.4322 | 0.7837 | 1.017 | 0.4225 | n.a. |
| 1f | 0.3968 | 0.8078 | 0.9770 | 0.4492 | n.a. |
| 1g | 1.398 | 0.2705 | **3.654** | **0.0217** | n.a. |
| 2a | 0.5769 | 0.6838 | **3.642** | **0.0289** | n.a. |
| 2b | 0.3039 | 0.8708 | **11.01** | **0.0002** | n.a. |
| 2c | 0.5353 | 0.712 | **5.109** | **0.0084** | n.a. |
| 2d | 2.418 | 0.0943 | **8.42** | **0.0009** | n.a. |
| 2g | 0.9522 | 0.4738 | 1.705 | 0.2248 | n.a. |
| 2h | 0.8162 | 0.5432 | 1.437 | 0.2917 | n.a. |
| 3a | 0.2799 | 0.8844 | **0.0359** | **0.1232** | n.a. |
| 3b | 0.4049 | 0.8011 | **4.943** | **0.0185** | n.a. |
| 3c | 1.106 | 0.4057 | 1.111 | 0.4037 | n.a. |
| 3d | 1.080 | 0.4035 | 0.525 | 0.7192 | n.a. |
| 3e | 0.9667 | 0.467 | 3.435 | 0.0516 | n.a. |
| 3g | 2.463 | 0.0901 | 0.288 | 0.8812 | n.a. |
| 3f | 1.198 | 0.3699 | 2.961 | 0.0746 | n.a. |
| 4a | 0.3651 | 0.8281 | 1.23 | 0.3581 | n.a. |
| 4b | n.a. | n.a. | n.a. | n.a. | n.a. |
| 4c | 0.4318 | 0.7829 | **7.125** | **0.0056** | **<0.0001** |
| 4d | n.a. | n.a. | n.a. | n.a. | n.a. |
| 4e | 0.3125 | 0.8632 | 1.024 | 0.4408 | n.a. |
| 6a | 0.5144 | 0.7272 | 0.1873 | 0.9397 | n.a. |
| 6b | n.a. | n.a. | n.a. | n.a. | n.a. |
| 6c | 0.8784 | 0.5103 | 3.007 | 0.0719 | n.a. |
| 6d | n.a. | n.a. | n.a. | n.a. | n.a. |
| 6e | 0.5442 | 0.7074 | 1.555 | 0.2598 | n.a. |
| 8a | 1.340 | 0.3008 | **3.557** | **0.0312** | n.a. |
| 8b | 0.7566 | 0.5693 | **3.297** | **0.0398** | n.a. |
| 8c | 1.617 | 0.2215 | 1.657 | 0.212 | n.a. |
| 8d | **1.297** | **0.3154** | 2.234 | 0.1142 | n.a. |

Compared to Untreated control with 3 mM $H_2O_2$

| | Brown-Forsythe test | | One-way ANOVA | | 6 μM ARI *vs* 6 μM OLA |
|---|---|---|---|---|---|
| Fig | F | *p*-value | F | *p*-value | *p*-value |
| 1a | n.a. | n.a. | n.a. | n.a. | n.a. |
| 1b | 0.1528 | 0.9574 | **7.788** | **0.0041** | n.a. |
| 1c | 0.0393 | 0.9968 | **10.55** | **<0.0001** | **<0.0001** |
| 1d | 0.9716 | 0.4518 | **3.480** | **0.0335** | n.a. |
| 1e | 1.788 | 0.1709 | **13.93** | **<0.0001** | n.a. |
| 1f | 0.6809 | 0.6159 | **3.691** | **0.0277** | n.a. |
| 1g | 0.4706 | 0.7567 | 1.723 | 0.1844 | n.a. |
| 2a | 0.6201 | 0.6551 | **5.013** | **0.0091** | n.a. |
| 2b | 1.620 | 0.2207 | **12.03** | **0.0001** | n.a. |
| 2c | 1.485 | 0.2562 | **6.397** | **0.0033** | n.a. |
| 2d | 0.3037 | 0.8706 | 2.888 | 0.0618 | n.a. |
| 2g | 0.1883 | 0.9391 | 1.564 | 0.2576 | n.a. |
| 2h | 0.6043 | 0.6684 | 1.833 | 0.1993 | n.a. |
| 3a | 0.2033 | 0.9308 | **3.895** | **0.0369** | n.a. |
| 3b | n.a. | n.a. | n.a. | n.a. | n.a. |
| 3c | 0.2113 | 0.9262 | 0.4404 | 0.777 | n.a. |
| 3d | 0.6302 | 0.649 | **7.687** | **0.0017** | **0.0013** |
| 3e | 0.4981 | 0.738 | **4.765** | **0.0206** | n.a. |
| 3g | 0.8613 | 0.5093 | **3.263** | **0.041** | n.a. |
| 3f | 0.2144 | 0.9244 | **6.099** | **0.0094** | **0.0009** |
| 4a | n.a. | n.a. | n.a. | n.a. | n.a. |
| 4b | 0.8447 | 0.5279 | **3.484** | **0.0498** | **0.0384** |
| 4c | n.a. | n.a. | n.a. | n.a. | n.a. |
| 4d | 1.015 | 0.4447 | **20.48** | **<0.0001** | **<0.0001** |
| 4e | 0.3989 | 0.8052 | **7.883** | **0.0039** | n.a. |
| 6a | n.a. | n.a. | n.a. | n.a. | n.a. |
| 6b | 0.8955 | 0.5017 | 3.222 | 0.0607 | **0.0241** |
| 6c | n.a. | n.a. | n.a. | n.a. | n.a. |
| 6d | 0.0855 | 0.985 | **6854** | **0.0064** | **0.0145** |
| 6e | 0.3925 | 0.8096 | **11.51** | **0.0009** | **0.0158** |
| 8a | 1.606 | 0.2243 | **6.74** | **0.0026** | **0.0422** |
| 8b | 1.196 | 0.3529 | **4.035** | **0.0204** | n.a. |
| 8c | 0.5342 | 0.7128 | **7.316** | **0.0018** | n.a. |
| 8d | 4.915 | 0.0098 | 1.19 | 0.3551 | n.a. |

# Supplementary Table S2

Compared to Baseline

| | Brown-Forsythe test | | One-way ANOVA | | 6 μM ARI *vs* 6 μM OLA |
|---|---|---|---|---|---|
| Fig | F | *p*-value | F | *p*-value | *p*-value |
| 2e | 0.3343 | 0.9527 | **50.29** | **<0.0001** | **n.a.** |
| 2f | 1.322 | 0.2868 | **165.5** | **<0.0001** | n.a. |

Compared to STS

| | Brown-Forsythe test | | One-way ANOVA | | 6 μM ARI *vs* 6 μM OLA |
|---|---|---|---|---|---|
| Fig | F | *p*-value | F | *p*-value | *p*-value |
| 2e | 0.1797 | 0.9437 | **12.56** | **0.0007** | n.a. |
| 2f | 0.6113 | 0.6640 | 9.812 | 0.0017 | n.a. |

# Supplementary Table S3

Baseline and 1.5 mM $H_2O_2$

| | Two-way ANOVA | | JNK 54: 6 μM ARI *vs* 6 μM OLA | JNK 46: 6 μM ARI *vs* 6 μM OLA |
|---|---|---|---|---|
| Fig | F | *p*-value | *p*-value | *p*-value |
| 5a | **4.382** | **0.0005** | n.a. | **0.002** |
| 5c | **76.18** | **<0.0001** | **<0.0001** | **<0.0001** |
| | **Two-way ANOVA** | | **ERK 44: 6 μM ARI *vs* 6 μM OLA** | **ERK 42: 6 μM ARI *vs* 6 μM OLA** |
| Fig | F | *p*-value | *p*-value | *p*-value |
| 5e | **2.344** | **0.0314** | n.a. | n.a. |
| 5g | **150** | **<0.0001** | **<0.0001** | **<0.0001** |

Baseline and 3 mM $H_2O_2$

| | Two-way ANOVA | | JNK 54: 6 μM ARI *vs* 6 μM OLA | JNK 46: 6 μM ARI *vs* 6 μM OLA |
|---|---|---|---|---|
| Fig | F | *p*-value | *p*-value | *p*-value |
| 5a | **3.898** | **0.0013** | **0.0097** | n.a. |
| 5d | **46.57** | **<0.0001** | **0.0082** | **<0.0001** |
| | **Two-way ANOVA** | | **ERK 44: 6 μM ARI *vs* 6 μM OLA** | **ERK 42: 6 μM ARI *vs* 6 μM OLA** |
| Fig | F | *p*-value | *p*-value | *p*-value |
| 5f | **6.114** | **<0.0001** | **0.0429** | **0.0452** |
| 5h | **32.17** | **<0.0001** | **<0.0001** | **<0.0001** |

# Supplementary Table S4

Compared to Baseline

| | t-test |
|---|---|
| Fig | *p*-value |
| 7a | 0.1097 |
| 7b | 0.9706 |
| 7c | **0.0109** |
| 7d | **0.0076** |
| 7e | **0.0147** |
| 7f | 0.0579 |

Compared to Untreated control with 1.5 mM $H_2O_2$

| | t-test |
|---|---|
| Fig | *p*-value |
| 7a | 0.4485 |
| 7b | 0.6448 |
| 7c | 0.0531 |
| 7d | 0.7527 |
| 7e | **0.0314** |
| 7f | **0.0004** |

Compared to Untreated control with 3 mM $H_2O_2$

| | t-test |
|---|---|
| Fig | *p*-value |
| 7a | 0.3043 |
| 7b | 0.4713 |
| 7c | **0.0088** |
| 7d | **0.0277** |
| 7e | 0.7953 |
| 7f | 0.5668 |

# Supplementary Table S5

Supplemental figure

| | Brown-Forsythe test | | ANOVA | | EBSS 1h *vs* EBSS 4h | EBSS 2h *vs* EBSS 4h |
|---|---|---|---|---|---|---|
| **Fig** | **F** | ***p*-value** | **F** | ***p*-value** | ***p*-value** | ***p*-value** |
| **S3a** | 0.4208 | 0.6659 | **9.505** | **0.0034** | **0.0038** | **0.0159** |
