# Supplementary figures and images for: Aripiprazole, but Not Olanzapine, Alters the Response to Oxidative Stress in Fao Cells by Reducing the Activation of Mitogen-Activated Protein Kinases (MAPKs) and Promoting Cell Survival
Source: Int J Mol Sci. 2024 Oct 16;25(20):11119. doi: 10.3390/ijms252011119 (PMC11508229; doi:10.3390/ijms252011119)

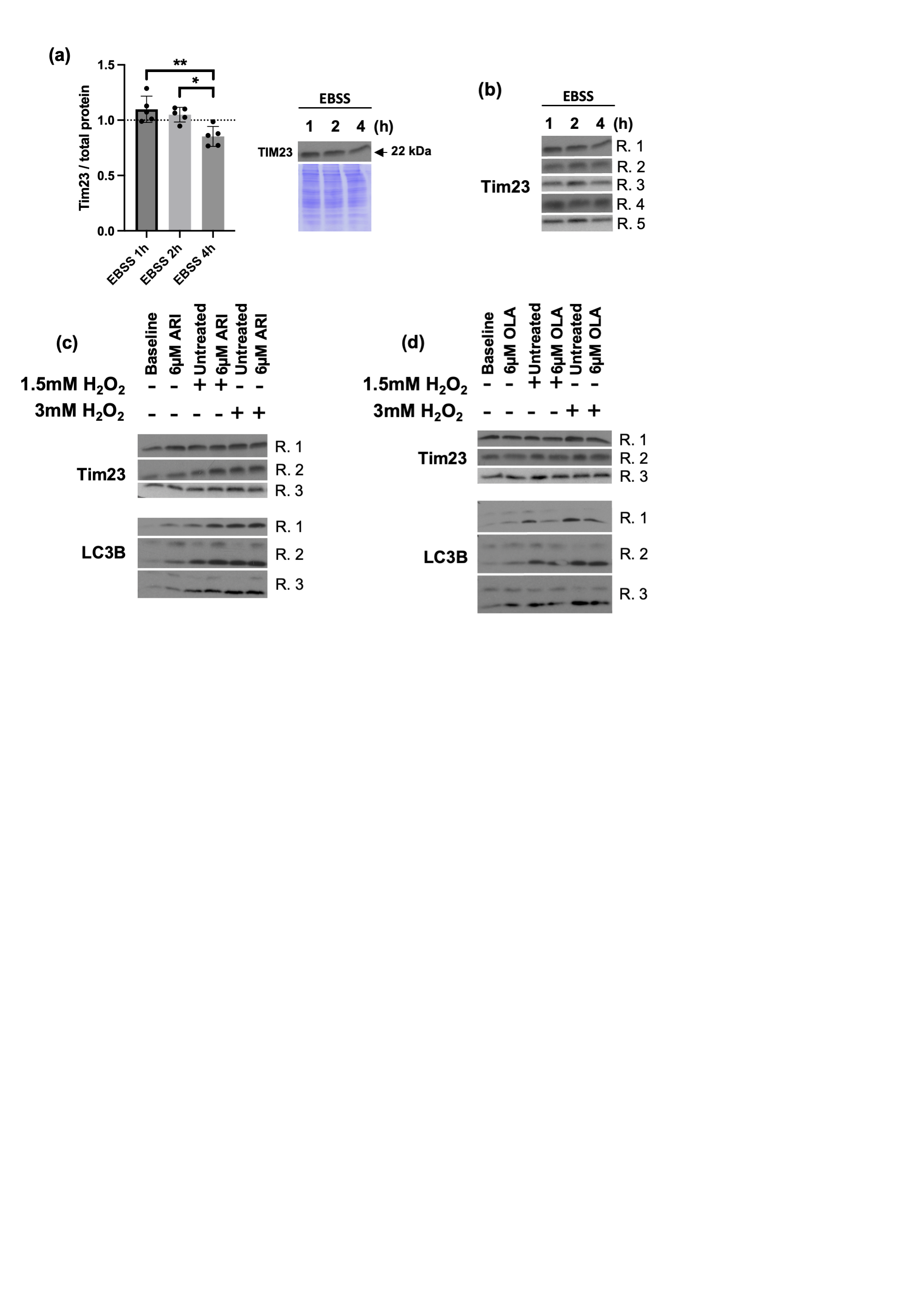

Supplement: Supplementary file 1 [file ijms-25-11119-s001.zip › FigureS1_BK_IM 20240820.tiff]

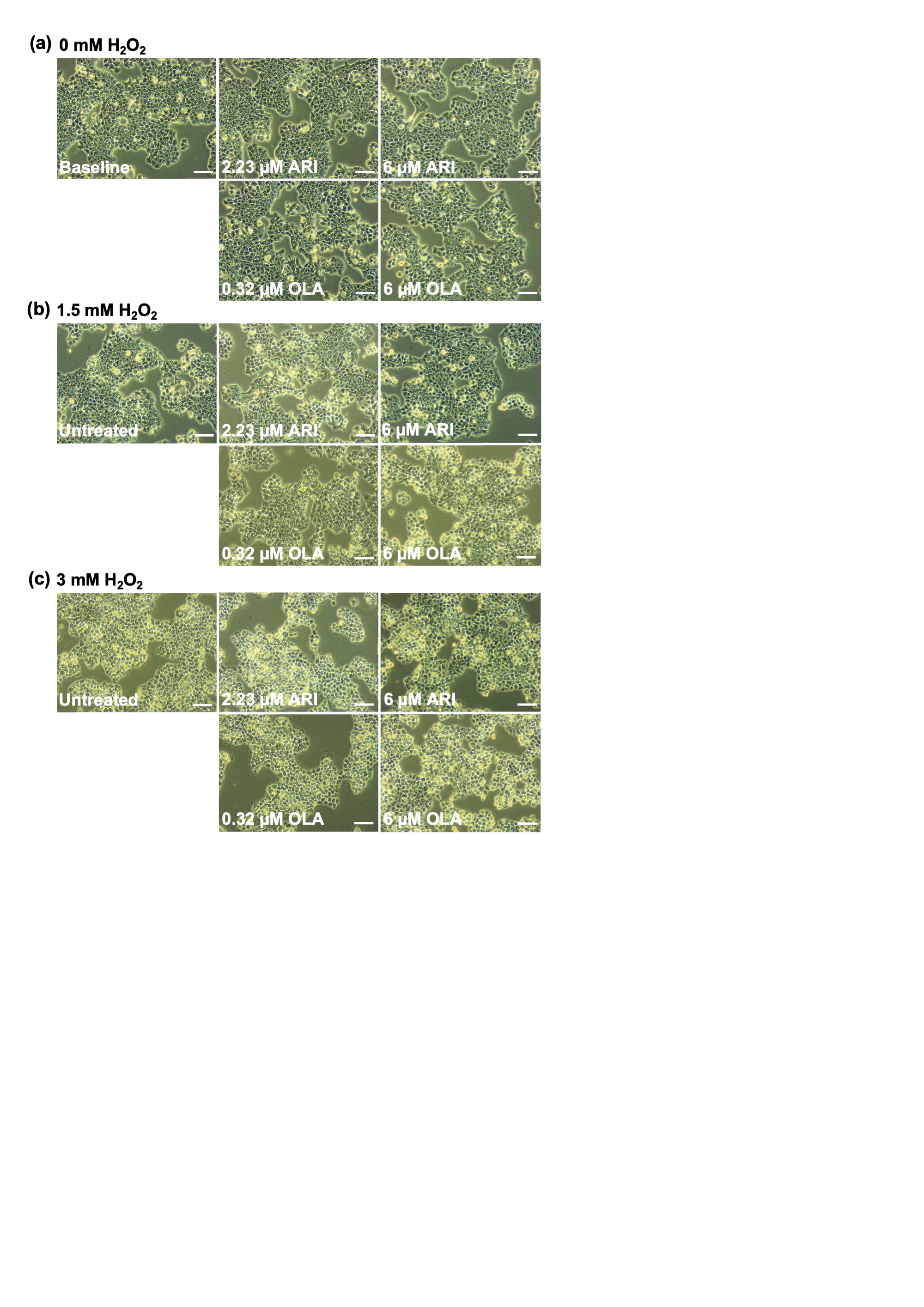

Supplement: Supplementary file 1 [file ijms-25-11119-s001.zip › FigureS2_BK_IM 20240820.tiff]

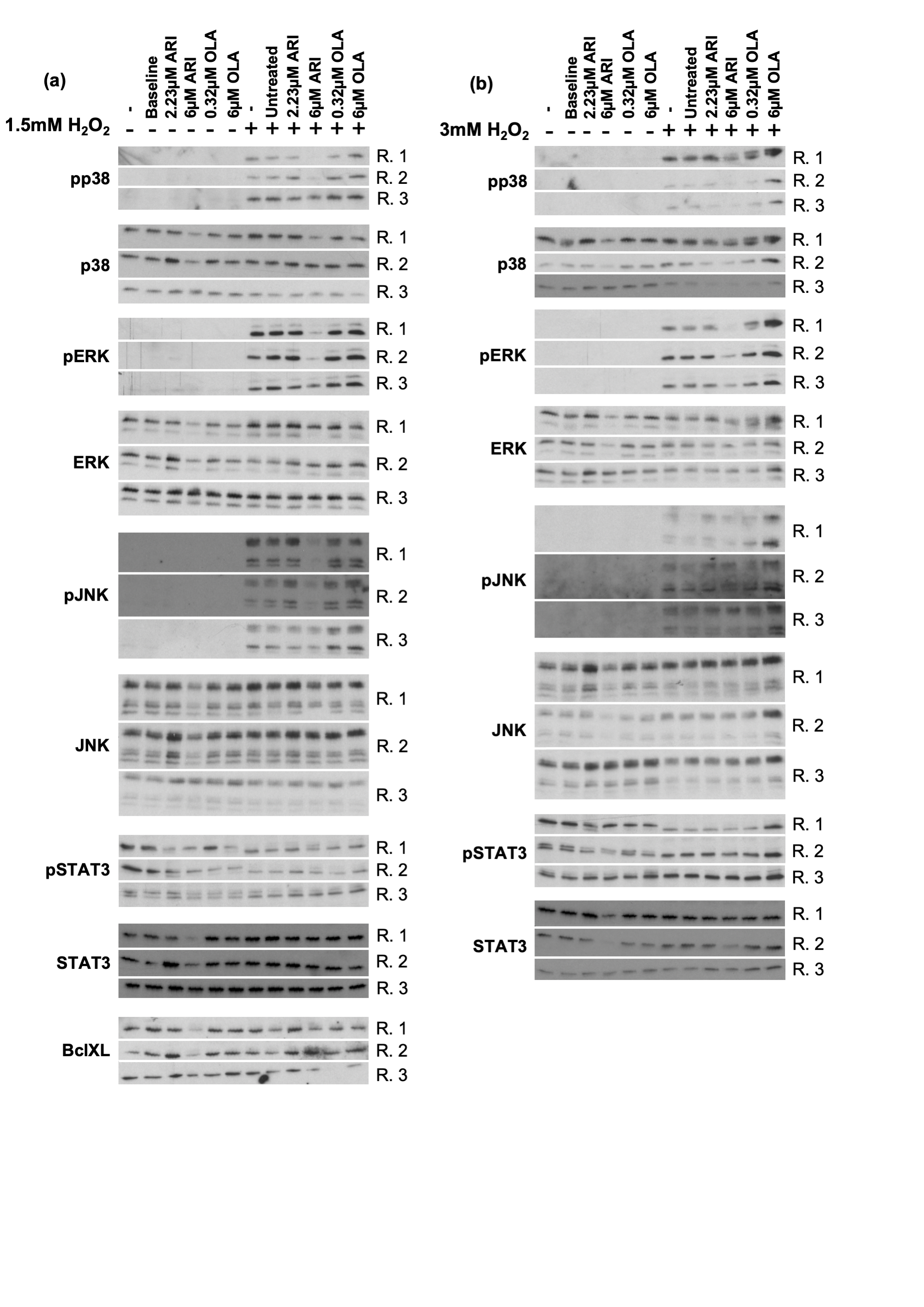

Supplement: Supplementary file 1 [file ijms-25-11119-s001.zip › FigureS3_BK_IM 20240820.tiff]

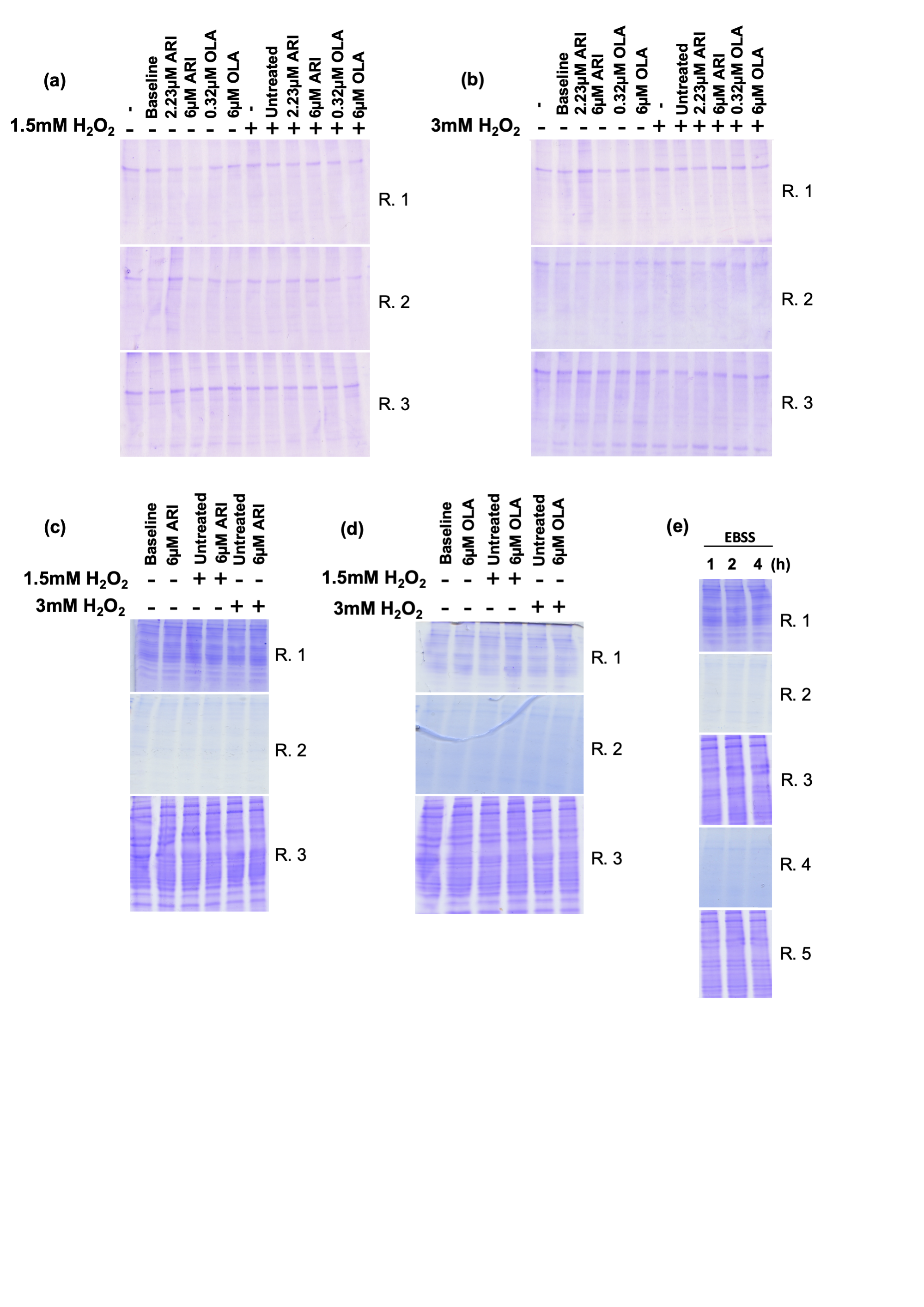

Supplement: Supplementary file 1 [file ijms-25-11119-s001.zip › FigureS4_BK_IM 20240820.tiff]
